# Indole-carbohydrazide linked phenoxy-1,2,3-triazole-*N*-phenylacetamide derivatives as potent α-glucosidase inhibitors: design, synthesis, in vitro α-glucosidase inhibition, and computational studies

**DOI:** 10.1186/s13065-023-00971-w

**Published:** 2023-06-15

**Authors:** Mehdi Emadi, Fahimeh Mosavizadeh-Marvest, Ali Asadipour, Yaghoub Pourshojaei, Samanesadat Hosseini, Somayeh Mojtabavi, Mohammad Ali Faramarzi, Bagher Larijani, Maryam Mohammadi-Khanaposhtani, Mohammad Mahdavi

**Affiliations:** 1 Electrical and Computer Engineering Department, Babol Noshirvani University of Technology, Babol, Iran; 2 Department of Medicinal Chemistry, Faculty of Pharmacy & Pharmaceutics Research Center, Institute of Neuropharmacology, Kerman University of Medical Sciences, Kerman, Iran; 3 Extremophile and Productive Microorganisms Research Center, Kerman University of Medical Sciences, Kerman, Iran; 4 Shahid Beheshti University of Medical Sciences, Tehran, Iran; 5 Department of Pharmaceutical Biotechnology, Faculty of Pharmacy, Tehran University of Medical Sciences, Tehran, Iran; 6 Endocrinology and Metabolism Research Center, Endocrinology and Metabolism Clinical Sciences Institute, Tehran University of Medical Sciences, Tehran, Iran; 7 Cellular and Molecular Biology Research Center, Health Research Institute, Babol University of Medical Sciences, Babol, Iran

**Keywords:** Indole, Carbohydrazide, 1,2,3-Triazole, α-Glucosidase, *N*-phenylacetamide

## Abstract

**Background:**

A new series of indole-carbohydrazide-phenoxy-1,2,3-triazole-*N*-phenylacetamide hybrids **11a–o** was designed based on molecular hybridization of the active pharmacophores of the potent α-glucosidase inhibitors. These compounds were synthesized and evaluated against α-glucosidase.

**Methods:**

The 15 various derivatives of indole-carbohydrazide-phenoxy-1,2,3-triazole-*N*-phenylacetamide scaffold were synthesized, purified, and fully characterized. These derivatives were evaluated against yeast α-glucosidase in vitro and in silico. ADMET properties of the most potent compounds were also predicted.

**Results:**

All new derivatives **11a–o** (IC_50_ values = 6.31 ± 0.03–49.89 ± 0.09 µM) are excellent α-glucosidase inhibitors in comparison to acarbose (IC_50_ value = 750.0 ± 10.0 µM) that was used as a positive control. Representatively, (*E*)-2-(4-((4-((2-(1*H*-indole-2-carbonyl)hydrazono)methyl) phenoxy)methyl)-1*H*-1,2,3-triazol-1-yl)-*N*-(4-methoxyphenyl)acetamide **11d** with IC_50_ = 6.31 µM against MCF-7 cells, was 118.8-times more potent than acarbose. This compound is an uncompetitive inhibitor against α-glucosidase and showed the lowest binding energy at the active site of this enzyme in comparison to other potent compounds. Furthermore, computational calculations predicted that compound **11d** can be an orally active compound.

**Conclusion:**

According to obtained data, compound **11d** can be a valuable lead compound for further structural development and assessments to obtain effective and potent new α-glucosidase inhibitors.

**Supplementary Information:**

The online version contains supplementary material available at 10.1186/s13065-023-00971-w.

## Introduction

Diabetes mellitus (DM) is a set of metabolic disease characterized by hyperglycemia [1]. This disorder mainly induced by the deficiency of insulin production or function [2]. DM led to vessels damages and becomes the cause of failure in many organs such as heart, kidneys, nerves, and the eyes [3]. The most common type of DM is type 2 diabetes (T2DM), which is usually associated with incomplete function of insulin [4]. According to the World Health Organization reports, the number of patients with T2DM will reach 360 million by 2030. The essential goal of treatment of T2DM is control of blood glucose level and the main drugs for this disease are oral blood glucose-lowering medications that acted with mechanisms such as augmentation of glucosuria, enhancement in the effect of insulin, reduction in resistance to insulin, and reduction in intestinal glucose absorption [5]. In order to decrease in intestinal glucose absorption, it is necessary to prevent the breakdown of carbohydrates into glucose by inhibition of carbohydrase intestinal enzymes such as α-glucosidase [6]. The commercially available α-glucosidase inhibitors are acarbose, voglibose, and miglitol [7]. Some studies showed that use of these drugs is associated with gastrointestinal complications including diarrhea, bloating, and abdominal panic [8]. Therefore, development of new efficient, safe, and potent α-glucosidase inhibitors is an attractive goal to medicinal chemists [9].

**Fig. 1:**
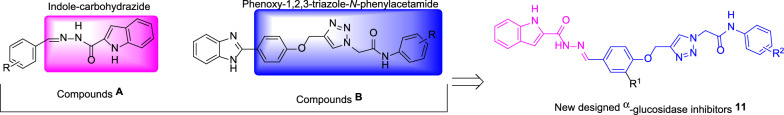
Design strategy for the new α-glucosidase inhibitors **11a–o**

Indole is a bicyclic *N*-heterocycle with various biological effects such as antimicrobial, antiviral, anticancer, anticonvulsant, antitubercular, and antimalarial properties [10–14]. In addition to, this ring has attracted much attention as a privileged pharmacophore for design of new potent α-glucosidase inhibitors [15]. During the recent years, several series of natural and synthetic indole derivatives with significant anti-α-glucosidase activity have been reported [15]. One of this effective synthetic series are indole-carbohydrazide derivatives **A** that most potent compound in this series inhibited α-glucosidase around 394-fold more that positive control acarbose (Fig. 1) [16]. Another *N*-heterocycle core that has recently received attention in the design of new α-glucosidase inhibitors is 1,2,3-triazole ring. Recently, numerous 1,2,3-triazole derivatives with significant inhibitory activities against α-glucosidase have been reported [17]. Moreover, combination of 1,2,3-triazole ring to phenoxy group and *N*-phenylacetamid derivatives led to the introduction of new potent α-glucosidase inhibitors containing phenoxy-1,2,3-triazole-*N*-phenylacetamide moiety [18]. As can be seen Fig. 1, the latter moiety is observed in potent α-glucosidase inhibitors **B** (IC_50_ values = 25.2 ± 0.9–176.5 ± 6.7 μM comparing with acarbose, 750.0 ± 12.5 μM) [19].

Based on the reported potent α-glucosidase inhibitors **A** and **B**, we designed indole-carbohydrazide-phenoxy-1,2,3-triazole-*N*-phenylacetamides **11** as new potent α-glucosidase inhibitors (Fig. 1). Therefore, we synthesized fifteen derivatives **11a–o** and evaluate their in vitro α-glucosidase inhibitory activities. Furthermore, in vitro kinetic analysis and in silico molecular docking study were also used to elucidate the interaction between the newly synthesized compounds and α-glucosidase. Moreover, pharmacokinetic and toxicity of these compounds were predicted by a reliable online software.

## Results and discussion

### Chemistry

**Scheme 1:**
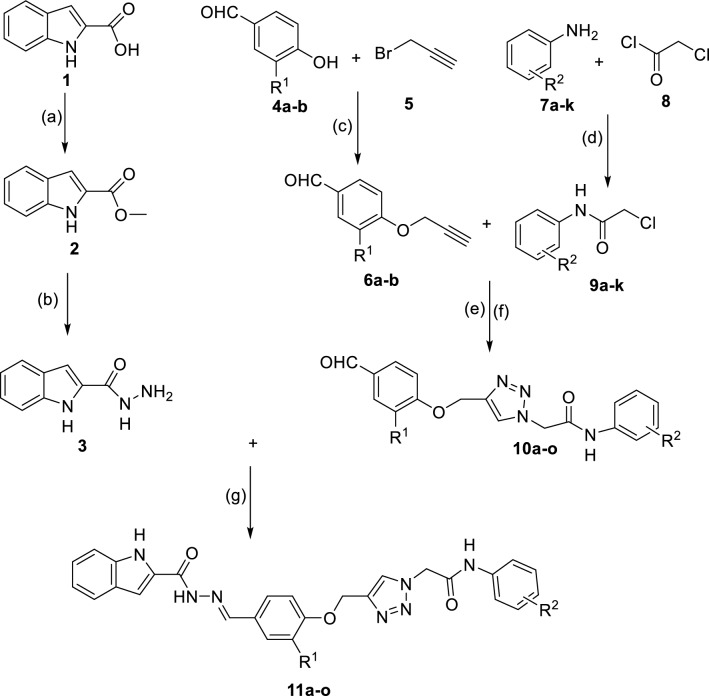
Synthetic routes to the new compounds **10a–o**: **a** H_2_SO_4_, Methanol, Reflux, 12 h; **b** Hydrazine, Ethanol, RT, 6 h; **c** K_2_CO_3_, DMF, room temperature, 3 h; **d** DMF, RT, 30 min; **e** NaN_3_, NEt_3_, H_2_O/t-BuOH, 1 h; **f** CuSO_4_·5H_2_O, sodium ascorbate, RT, 24–48 h. **g** Acetic acid, Ethanol, RT, 6 h

The desired indole-carbohydrazide-phenoxy-1,2,3-triazole-*N*-phenylacetamide derivatives **11a–o** were prepared in good yields as shown in Scheme 1. The preparation of the target compounds **11a–o** was started from conversion of indole-2-carboxylic acid **1** to methyl 1*H*-indole-2-carboxylate **2** in the presence of sulfuric acid in methanol. The obtained compound **2** was converted to 1*H*-indole-2-carbohydrazide **3** in the presence of hydrazine in ethanol. On the other hand, 4-(prop-2-ynyloxy)benzaldehydes **6a–b** were produced by a reaction between 4-hydroxy-benzaldehyde derivatives **4a–b** and propargyl bromide **5** in the presence of K_2_CO_3_ in DMF at room temperature. *N*-phenyl-2-chloroacetamide derivatives **9a–k** were prepared by the reaction between aniline derivatives **7a–k** and chloroacetyl chloride **8** in DMF at room temperature. Compounds **6a–b** and **9a–k** produced compounds **10a–o** by the following reactions: various chloride derivatives **9a–k** and sodium azide were reacted in the mixture of H_2_O and t-BuOH (1:1) in the presence of Et_3_N at room temperature and after that, a mixture of compounds **6a–b**, sodium ascorbate, and CuSO_4_·5H_2_O was added to azide mixture and the reaction was continued at room temperature to afford the 1,2,3-triazole derivatives **10a–o**. Finally, 1*H*-indole-2-carbohydrazide **3** was reacted with 1,2,3-triazole derivatives **10a–o** in the presence of acetic acid in ethanol to give target compounds **11a–o**. Investigation of the spectroscopy data of new compounds **11a–o** revealed that these compounds exist only in an isomeric form. On the other hand, stable isomeric form of these compounds is E-form [20]. Therefore, these compounds exist in the stable E-isomer form.

### In vitro anti-α-glucosidase activity and SAR discussion

The in vitro anti-α-glucosidase activity of the new indole-carbohydrazide-phenoxy-1,2,3-triazole-*N*-phenylacetamide derivatives **11a–o** was evaluated by a standard method [21]. Acarbose was chosen as positive control and enzyme inhibition activities are expressed as IC_50_ values in Table 1. As evidenced by obtained IC_50_ values, both phenoxy derivatives **11a–h** and 2-methoxyphenoxy derivatives **11i–o** showed excellent anti-α-glucosidase activity. These compounds with IC_50_ values ranging from 6.31 ± 0.03 to 49.89 ± 0.09 μM are promising α-glucosidase inhibitors when compared to acarbose with IC_50_ value of 750.0 ± 10.0 μM.

**Table 1:** Structures and anti-α-glucosidase activities (IC_50_ values, µM) of indole-carbohydrazide-phenoxy-1,2,3-triazole-*N*-phenylacetamide derivatives **11a–o **
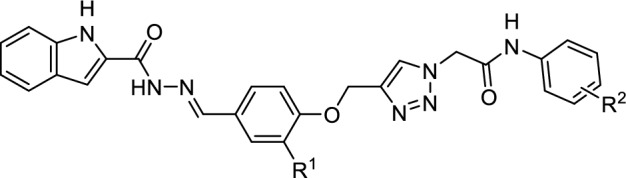

Compound	R^1^	R^2^	IC_50_ (µM)
**11a**	H	H	15.21 ± 0.18
**11b**	H	2-CH_3_	8.88 ± 0.07
**11c**	H	4-CH_3_	19.12 ± 0.08
**11d**	H	4-OCH_3_	6.31 ± 0.03
**11e**	H	2-Cl	26.97 ± 0.25
**11f**	H	4-Cl	9.53 ± 0.12
**11g**	H	4-Br	49.89 ± 0.09
**11h**	H	4-NO_2_-3-CH_3_	35.11 ± 0.07
**11i**	OCH_3_	H	11.25 ± 0.15
**11j**	OCH_3_	4-OCH_3_	24.19 ± 0.1
**11k**	OCH_3_	4-Cl	31.56 ± 0.12
**11l**	OCH_3_	2,3-Dichloro	18.2 ± 0.81
**11m**	OCH_3_	3,5-Dichloro	14.2 ± 0.21
**11n**	OCH_3_	4-Br	8.3 ± 0.16
**11o**	OCH_3_	2-Cl-3-NO_2_	12.67 ± 0.05
Acarbose	–	-	750.0 ± 10.0

Among the synthesized compounds, the *N*-4-methoxyphenylacetamide derivative **11d** from phenoxy series was the most potent entry. The latter compound was 118.8 times more potent than acarbose. Replacement of methoxy substituent of compound **11d** (IC_50_ = 6.31 µM) with chloro, methyl, and or bromo substituents, as in the cases of compounds **11f** (IC_50_ = 9.53 µM), **11c** (IC_50_ = 19.12 µM), and **11 g** (IC_50_ = 49.89 µM), diminished the inhibitory activity to 1.5, 3, and 8 folds, respectively. A survey on the structures and inhibitory activities in Table 1 demonstrated that in phenoxy series (compounds **11a–h**), the chloro substituent had the best effect in 4-position of *N*-arylacetamide moiety (compound **11f** with IC_50_ = 9.53 µM vs. compound **11e** with IC_50_ = 26.97 µM) while methyl substituent had the best effect in 2-position (compound **11b** with IC_50_ = 8.88 µM vs. compound **11c** with IC_50_ = 19.12 µM).

**Fig. 2:**
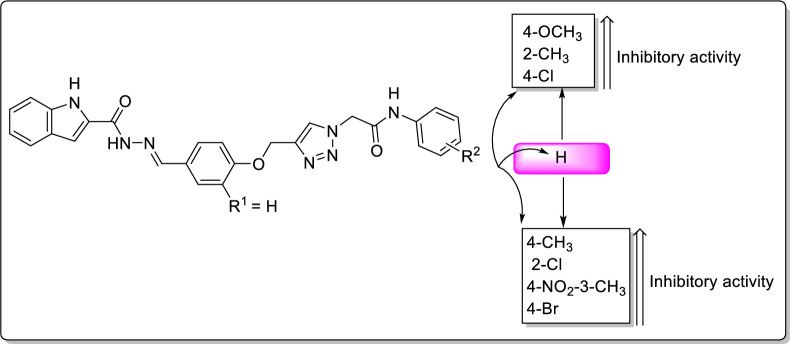
The comparison of inhibitory activity of un-substituted compound **11a** with substituted compounds **11b–h** of the phenoxy series

Moreover, the comparison of inhibitory activity of the substituted compounds **11b–h** with un-substituted compounds **11a** was depicted in Fig. 2. This figure showed that introduction of 4-methoxy, 2-methyl or 4-chloro on phenyl ring of *N*-arylacetamide moiety improved inhibitory activity in comparison to un-substituted compound **11a** while the presence of 4-methyl, 2-chloro, 4-nitro-3-methyl, and or 4-bromo substituent deteriorated inhibitory activity. The weakest inhibitor in this series was 4-bromo derivative **11g**.

**Fig. 3:**
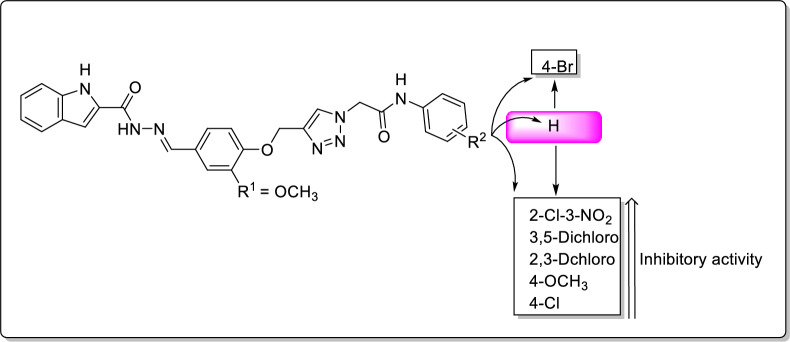
The comparison of inhibitory activity of un-substituted compound **11i** with substituted compounds **11j–o** in the 2-methoxyphenoxy series

Unlike the phenoxy series, in the 2-methoxyphenoxy series (compounds **11i–o**), the most potent compound was 4-bromo derivative **11n**. The second potent compound in this series was un-substituted compound **11i** and introduction of 2-Cl-3-NO_2_, 3,5-dichloro, 2,3-dichloro, 4-OCH_3_ or 4-Cl led to a decrease in the inhibitory activity (Fig. 3). The weakest inhibitor in 2-methoxyphenoxy series was 4-chloro derivative **11g**.

The comparison of IC_50_ values of phenoxy derivatives with their corresponding 2-methoxyphenoxy analogs revealed that phenoxy analogs with 4-methoxy (compound **11d**) and 4-chloro (compound **11f**) were more active than their 2-methoxyphenoxy analogs (compounds **11j** and **11k**, respectively) while 2-methoxyphenoxy analogs with un-substituented phenyl (compound **11i**) and 4-bromophenyl (compound **11n**) were more potent of their corresponding analogs of phenoxy series (compounds **11a** and **11g**, respectively).

**Fig. 4:**
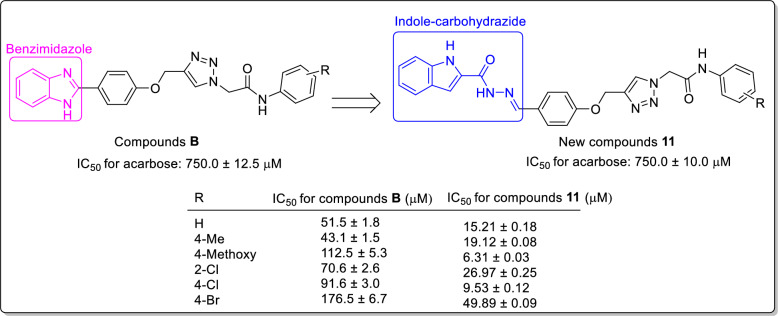
α-Glucosidase inhibitory activities of benzimidazole-phenoxy-1,2,3-triazole-*N*-phenylacetamides **B** and their corresponding analogs of new compounds **11**

On the other hand, the comparison of IC_50_ values of the new indole-carbohydrazide-phenoxy-1,2,3-triazole-*N*-phenylacetamide derivatives **11** with their corresponding analogs of benzimidazole-phenoxy-1,2,3-triazole-*N*-phenylacetamide series **B** revealed that replacement of indole-carbohydrazide moiety instead of benzimidazole ring led to a significant increase in inhibitory activity as observed in Fig. 4 [19].

### Kinetic study

**Fig. 5:**
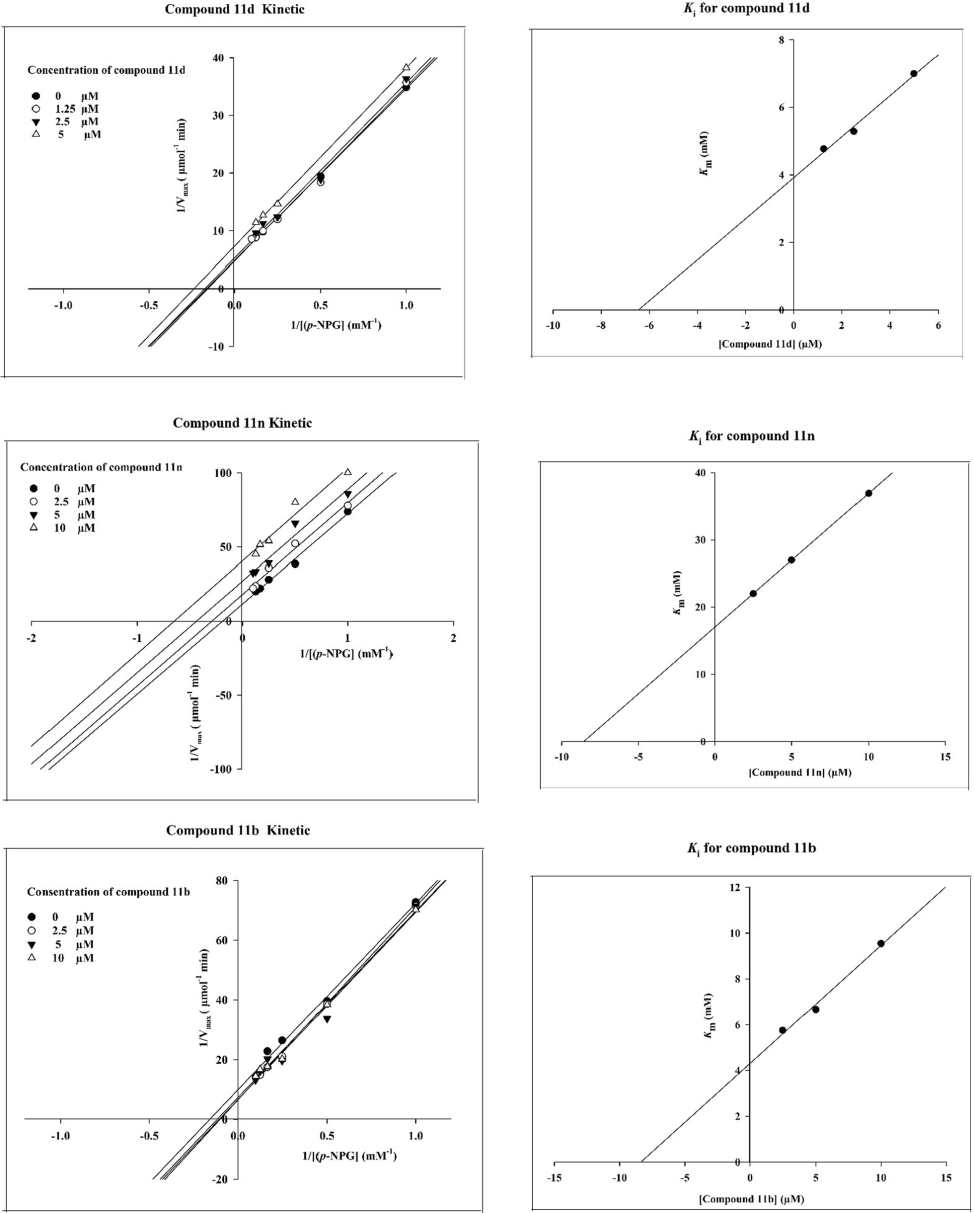
Kinetic studies of the most potent compounds **11d**, **11n**, and **11b**

To determine the mechanism of α-glucosidase inhibition of the newly synthesized compounds, the kinetic study was performed on the most potent compounds **11d**, **11n**, and **11b** as representative compounds. To construct the Lineweaver–Burk plots, enzymatic reactions were performed in the four concentrations of substrate (*p*-nitrophenyl-glucopyranoside in concentrations 1–4 mM) and inhibitor (compound **11d** at concentrations: 0, 1.25, 2.5 and 5 µM and compounds **11n** and **11b** at concentrations: 0, 2.5, 5 and 10 µM). Then, the Lineweaver-Burke plots were depicted using by the reciprocal of velocity and substrate concentration (Fig. 5). Lineweaver–Burk plots of the studied compounds demonstrated that in the presence of compounds **11d**, **11n**, and **11b** both V_max_ and K_m_ values were decreased. Therefore, the latter compounds are uncompetitive inhibitors. Using by the Lineweaver–Burk secondary plot (Fig. 5), K_i_ values of 6.3, 8.3, and 8.5 µM were determined for compounds **11d**, **11n**, and **11b**, respectively.

### Molecular modeling

Additionally to the SAR evaluation based on in vitro data, we performed in silico docking studies on the most potent compounds **11d** and **11n**. Crystal structure of the *Saccharomyces cerevisiae* α-glucosidase was not available in the protein data bank, thus, we used of a modeled enzyme based on previous work [21]. Docking study of positive control acarbose demonstrated that this drug formed following interactions in the α-glucosidase active with binding energy of − 4.04 kcal/mol:

1. Hydrogen bonds with Thr301, Gln322, Thr307, Arg312, Glu304, Ser308, Asn241
2. Non-classical hydrogen bond with His239
3. Hydrophobic interaction with His279
4. Unfavorable interactions with Arg312 and Thr207.

**Fig. 6:**
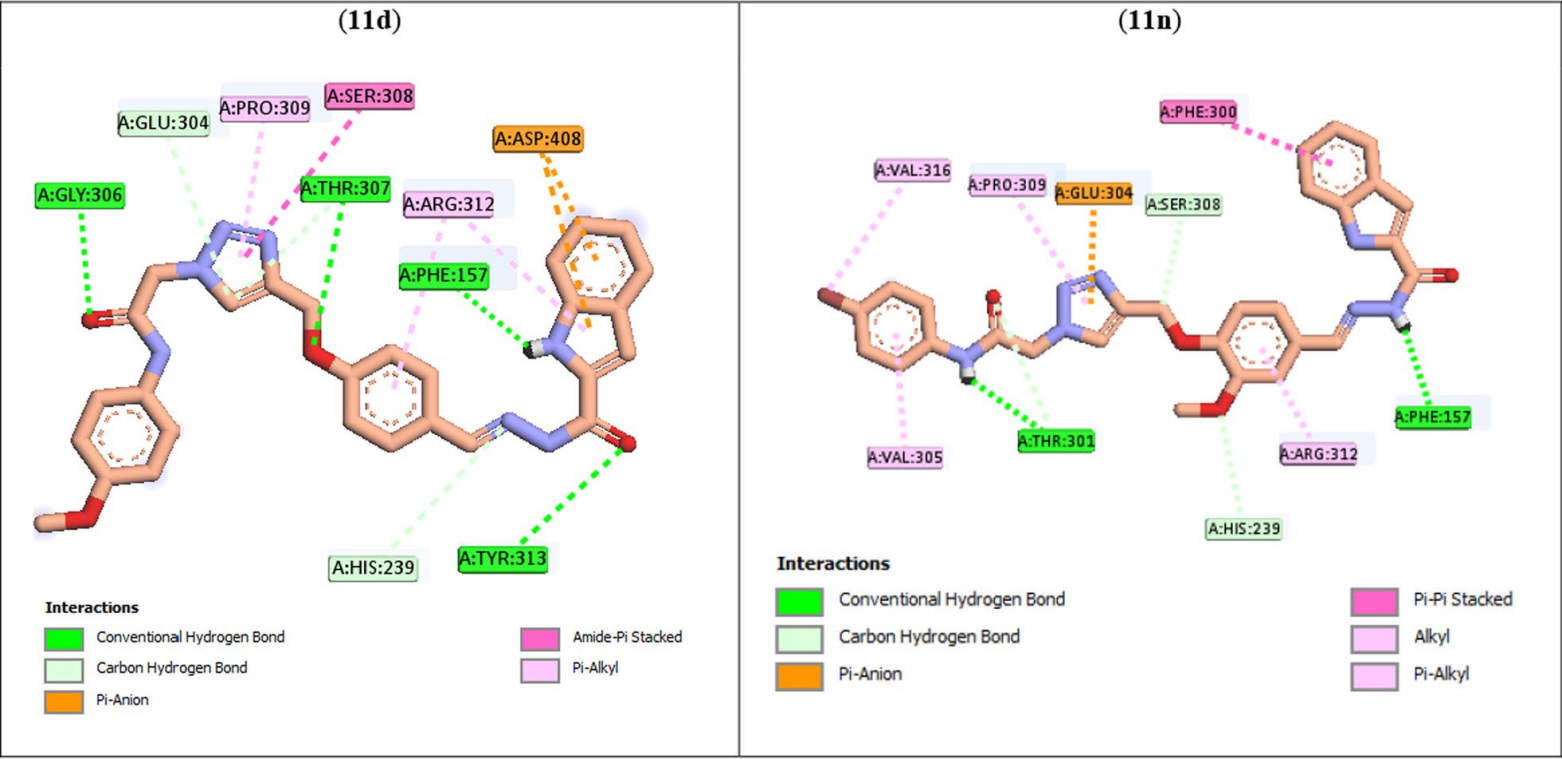
Interaction modes of the most potent compounds **11d** and **11n** in the active site of α-glucosidase

As can be seen Fig. 6, the most active derivative **11d** established hydrogen bonds with active site residues Gly306, Thr307, Phe157, and Tyr313. Compound **11d** formed two π–anion interactions with Asp408. Several hydrophobic interactions were also observed between compound **11d** and residues Pro309, Ser308, and Arg312. In addition, this compound formed three non-classical hydrogen bonds with Glu304, Thr307, and His239. Interaction mode of the second potent compound **11n** demonstrated that this compound formed classical hydrogen bonds with residues Thr301 and Phe157 and non-classical hydrogen bonds with Thr301, Ser308, and His239 (Fig. 6). Furthermore, this compound also created a π–anion interaction (Glu304) and several hydrophobic interactions (Val305, Val316, Pro309, Phe300, and Arg312) with the active site.

Binding energies, type of interactions, interacting unit of the compounds **11d** and **11n**, and involved amino acids in the interactions were listed in Table 2. As can be seen in this table, binding energy values of these compounds were lower than acarbose. On the other hand, the first potent compound **11d** had binding energy value lower than the second potent compound **11n**. These results are in agreement with the obtained results from in vitro enzymatic inhibition assay.

**Table 2:** Interaction mode details of the most potent compounds **11d** and **11n**

Compound	Binding energy	Interaction	Interacting unit of the ligand	Amino acid
**11d**	− 9.01	H-bond	NH–CO	Gly306
		Hydrophobic	1,2,3-triazol	Pro309
		Hydrophobic	1,2,3-triazol	Ser308
		H-bond	O of phenoxy linker	Thr307
		Hydrophobic	Phenyl of phenoxy linker	Arg312
		H-bond	C=N–NH–CO	Tyr313
		Hydrophobic	Indole	Arg312
		π–Anion	Indole	Asp408
**11n**	− 8.96	Hydrophobic	4-Br	Val316
		Hydrophobic	Pendant phenyl	Val305
		H-bond	NH–CO	Thr301
		Hydrophobic	1,2,3-triazol	Pro309
		π–Anion	1,2,3-triazol	Glu304
		Hydrophobic	Phenyl of phenoxy linker	Arg312
		H-bond	C=N–NH–CO	Phe157
		Hydrophobic	Indole	Phe300

### In vitro cytotoxicity assay

To assess of the cytotoxic effects of the new synthesized compounds, cytotoxicity of compounds **11d** and **11n** as the representative compounds was evaluated against the normal human dermal fibroblast (HDF) cell line [22]. Results revealed that these compounds were non-cytotoxic on the studied normal cell line at 50 μM concentration.

### In silico pharmacokinetic and toxicity studies

Pharmacokinetic and toxicity predictions of acarbose and the most potent compounds **11d** and **11n** were performed by PreADMET online software and the obtained results were listed in Table 3 [23]. As can be seen in this table, positive control acarbos did not follow of Lipinski ‘Rule of five’ while compounds **11d** and **11n** followed of this rule. Acarbose, **11d**, and **11n** had poor permeability to Caco-2. Permeability to blood brain barrier (BBB) and skin for these compounds is in the acceptable range. Compounds **11d** and **11n** had high human intestinal absorption (HIA) while acarbose had no HIA. In silico toxicity prediction demonstrated that acarbose and compound **11d** are mutagen while compound **11n** is not mutagen. Moreover, this study predicted that acarbose and compound **11d** had carcinogenic effect on mouse and did not have this effect on rat while compound **11n** has carcinogenic effect on rat and did not have carcinogenic effect mouse. Cardiotoxicity (hERG inhibition) of acarbose is ambiguous while compounds **11d** and **11n** in term of this type of toxicity have high risk.

**Table 3:** Druglikeness/ADME/T profile of acarbose and the most potent compounds **11d** and** 11n**

Druglikeness/ADME/T^a^	Compound		
	Acarbose	**11d**	**11n**
Rule of five	Violated	Suitable	Suitable
Caco2	9.44448	3.03438	18.8466
HIA	0.000000	91.952780	94.030638
BBB	0.0271005	0.272919	0.411249
Skin permeability	− 5.17615	− 3.52204	− 3.34921
Ames test	Mutagen	Mutagen	Non-mutagen
Carcino mouse	Positive	Positive	Negative
Carcino rat	Negative	Negative	Positive
hERG inhibition	Ambiguous	High risk	High risk

^a^The recommended ranges for Caco_2_: < 25 poor, > 500 great, HIA: > 80% is high < 25% is poor, BBB = − 3.0–1.2, and Skin_Permeability = − 8.0 to − 1.0

### Experimental

All chemicals were purchased from Sigma-Aldrich (USA) and were applied without further purification. Melting points of the new compounds **11a–o** were measured on an Electrothermal 9100 apparatus. Elemental analyses of the latter compounds for C, H and N were performed using a Heraeus CHN-O-Rapid analyzer. IR spectra of the title compounds were recorded on a Shimadzu IR-460 spectrometer. ^1^H and ^13^C NMR spectra were measured (DMSO-*d*_6_ solution) with Bruker DRX-300 (at 301 and 76 MHz) instrument.

### Synthesis of methyl 1*H*-indole-2-carboxylate 2

A mixture of indole-2-carboxylic acid **1** (10 mmol), sulfuric acid (2 mL), and methanol (20 mL) was heated under reflux condition for 12 h. Then, cold water was added to the latter mixture reaction and the obtained mixture was extracted by ethyl acetate. The ethyl acetate was evaporated under reduced pressure to produce pure methyl 1*H*-indole-2-carboxylate **2**.

### Synthesis of 1*H*-indole-2-carbohydrazide 3

A solution of methyl 1*H*-indole-2-carboxylate **2** (3 mmol) and hydrazine (9 mmol) in ethanol (20 mL) was stirred at room temperature for 6 h. Then, water was added to the mixture reaction and the white precipitate of 1*H*-indole-2-carbohydrazide **3** as final product in this step was appeared. This pure precipitate was separated by filtration.

### General procedure for the synthesis of 4-(prop-2-ynyloxy)benzaldehydes 6a–b

A suspension of 4-hydroxy-3-methoxybenzaldehydes **4a–b** (1 mmol) and K_2_CO_3_ (1 mmol) in DMF (10 mL) was stirred at room temperature for 1 h. After that, propargyl bromide **5** (1.2 mmol) in DMF (5 mL) in a dropwise manner was added to the latter suspension and the final reaction mixture was stirred at room temperature for 2 h. Then, this reaction mixture was poured into crushed ice and filtered off. The obtained residue was recrystallized in ethanol to give 3-methoxy-4-(prop-2-ynyloxy) benzaldehydes **6a–b**.

### General procedure for the synthesis of *N*-phenyl-2-chloroacetamide derivatives 9a–k

Aniline derivatives **7a–k** and chloroacetyl chloride **8** in DMF were stirred at room temperature for 30 min. At the end of the reaction (checked by TLC), the reaction mixture was diluted with cold water, poured into crushed ice, and the obtained white precipitates were filtered off. The residues were washed with water to obtain pure *N*-phenyl-2-chloroacetamides **9a–k**.

### General procedure for the synthesis of indole-carbohydrazide-phenoxy-1,2,3-triazole-*N*-phenylacetamide derivatives 11a–o

At first, 1,2,3-triazol derivatives **10a–o** were prepared. For this purpose, a mixture of *N*-phenyl-2-chloroacetamides **9a–k** (1.1 mmol), sodium azide (0.9 mmol), and Et_3_N (1.3 mmol) in the mixture of water plus t-BuOH (10 mL, 1:1) was stirred at room temperature for 1 h. Then, a mixture of 4-(prop-2-ynyloxy)benzaldehydes **6a–b** (1 mmol), sodium ascorbate, and CuSO_4_·5H_2_O (7 mol %) was added to the pervious mixture, and the final mixture was stirred at room temperature for 24–48 h. After completion of the reaction (monitored by TLC), the reaction mixture was diluted with cold water and poured into ice. Then, precipitated products **10a–o** were filtered off, washed with water, and purified by recrystallization in ethanol. Finally, a mixture of the latter compounds (1 mmol), 1*H*-indole-2-carbohydrazide **3** (1 mmol), and acetic acid (3 drops) in ethanol (20 mL) was stirred at room temperature for 6 h. After that, water was added to the latter mixture and pure products **11a–o** were filtered off (Additional file 1).

### (*E*)-2-(4-((4-((2-(1*H*-indole-2-carbonyl)hydrazono)methyl)phenoxy)methyl)-1*H*-1,2,3-triazol-1-yl)-*N*-phenylacetamide (11a)

White solid; isolated yield: 83%, mp: 194–196 °C; IR (KBr, υ): 3428, 3031, 1677 cm^−1^; ^1^H NMR (301 MHz, DMSO-*d*_6_) δ 11.83–11.68 (m, 2H, NH–N and NH of indole), 10.53 (s, 1H, NH–C=O), 8.46 (s, 1H, CH=N), 8.33 (s, 1H), 7.75 (d, *J* = 8.5 Hz, 2H), 7.63 (d, *J* = 7.4 Hz, 2H), 7.57 (d, *J* = 8.1 Hz, 1H), 7.51 (d, *J* = 8.1 Hz, 1H), 7.39–7.32 (m, 3H), 7.25–7.16 (m, 3H), 7.14–7.08 (m, 2H), 5.41 (s, 2H, CH_2_–C=O), 5.29 (s, 2H, O–CH_2_); ^13^C NMR (76 MHz, DMSO-*d*_6_) δ 164.65, 160.08, 158.00, 147.50, 142.72, 139.65, 138.89, 137.27, 130.68, 130.52, 129.39, 129.15, 128.63, 126.90, 126.00, 124.26, 122.22, 120.41, 115.60, 112.86, 103.93, 61.66, 52.74; Anal Calcd for C_27_H_23_N_7_O_3_, C, 65.71, H, 4.70, N, 19.87; Found: C, 65.72, H, 4.67, N, 19.80.

### (*E*)-2-(4-((4-((2-(1*H*-indole-2-carbonyl)hydrazono)methyl)phenoxy)methyl)-1*H*-1,2,3-triazol-1-yl)-*N*-(o-tolyl)acetamide (11b)

White solid; isolated yield: 89%, mp: 208–210 °C; IR (KBr, υ): 3281, 3057, 1666 cm^−1^; ^1^H NMR (301 MHz, DMSO-*d*_6_) δ 11.91–11.80 (m, 2H, NH–N and NH of indole), 10.45 (s, 1H, NH–C=O), 8.33 (s, 1H, CH= ), 7.71 (d, *J* = 8.0 Hz, 1H), 7.52 (d, *J* = 8.0 Hz, 3H), 7.47–7.40 (m, 1H), 7.40–7.34 (m, 1H), 7.32–7.22 (m, 3H), 7.21–7.00 (m, 4H), 5.39 (s, 2H, CH_2_–C=O), 5.26 (s, 2H, O-CH_2_), 2.27 (s, 3H, CH_3_); ^13^C NMR (76 MHz, DMSO-*d*_6_) δ 164.39, 162.77, 158.06, 149.77, 147.97, 137.30, 136.37, 133.26, 129.77, 128.64, 127.95, 127.52, 126.02, 124.29, 122.29, 122.22, 120.45, 119.73, 113.46, 112.97, 112.88, 108.88, 104.00, 55.88, 36.24, 20.92; Anal Calcd for C_28_H_25_N_7_O_3_, C, 66.26, H, 4.96, N, 19.32; Found: C, 66.27, H, 4.90, N, 19.37.

### (*E*)-2-(4-((4-((2-(1*H*-indole-2-carbonyl)hydrazono)methyl)phenoxy)methyl)-1*H*-1,2,3-triazol-1-yl)-*N*-(p-tolyl)acetamide (11c)

White solid; isolated yield: 93%, mp: 180–182 °C; IR (KBr, υ): 3400, 2962, 1681 cm^−1^; ^1^H NMR (301 MHz, DMSO-*d*_6_) δ 11.87–11.64 (m, 2H, NH–N and NH of indole), 10.44 (s, 1H, NH–C=O), 8.51–8.40 (m, 1H, CH=N), 8.33 (s, 1H), 7.79–7.68 (m, 3H), 7.54–7.48 (m, 3H), 7.39–7.32 (m, 1H), 7.26 (d, *J* = 7.0 Hz, 1H), 7.22 (d, *J* = 3.2 Hz, 1H), 7.19–7.06 (m, 4H), 5.39 (s, 2H, CH_2_–C=O), 5.29 (s, 2H, O–CH_2_), 2.27 (s, 3H, CH_3_); ^13^C NMR (76 MHz, DMSO-*d*_6_) δ 164.38, 160.09, 158.02, 147.49, 137.28, 136.37, 133.26, 130.76, 129.77, 129.16, 128.62, 127.69, 127.51, 126.90, 124.26, 122.21, 120.42, 119.73, 115.60, 112.87, 103.90, 61.66, 56.54, 20.92; Anal Calcd for C_28_H_25_N_7_O_3_, C, 66.26, H, 4.96, N, 19.32; Found: C, 66.20, H, 4.99, N, 19.37.

### (*E*)-2-(4-((4-((2-(1*H*-indole-2-carbonyl)hydrazono)methyl)phenoxy)methyl)-1*H*-1,2,3-triazol-1-yl)-*N*-(4-methoxyphenyl)acetamide (11d)

White solid; isolated yield: 90%, mp: 229–231 °C; IR (KBr, υ): 3455, 3143, 1680 cm^−1^; ^1^H NMR (301 MHz, DMSO-*d*_6_) δ 11.85–11.60 (m, 2H, NH–N and NH of indole), 10.38 (s, 1H, NH–C=O), 8.50–8.39 (m, 1H, CH=N), 8.32 (s, 1H), 7.80–7.68 (m, 3H), 7.56–7.49 (m, 3H), 7.38–7.31 (m, 1H), 7.28–7.18 (m, 3H), 7.10 (t, *J* = 8.0 Hz, 1H), 6.94 (d, *J* = 9.0 Hz, 2H), 5.37 (s, 2H, CH_2_–C=O), 5.29 (s, 2H, O–CH_2_), 3.75 (s, 3H, O–CH_3_); ^13^C NMR (76 MHz, DMSO-*d*_6_) δ 164.11, 160.09, 158.03, 156.05, 147.49, 142.71, 137.28, 131.98, 130.69, 129.16, 127.70, 127.53, 126.86, 124.26, 122.22, 121.30, 120.41, 115.61, 114.51, 112.87, 103.89, 61.68, 55.65, 52.67; Anal Calcd for C_28_H_25_N_7_O_4_, C, 64.24, H, 4.81, N, 18.73; Found: C, 64.20, H, 4.85, N, 18.75.

### (*E*)-2-(4-((4-((2-(1*H*-indole-2-carbonyl)hydrazono)methyl)phenoxy)methyl)-1*H*-1,2,3-triazol-1-yl)-*N*-(2-chlorophenyl)acetamide (11e)

White solid; isolated yield: 85%, mp > 250 °C; IR (KBr, υ): 3288, 3053, 1668 cm^−1^; ^1^H NMR (301 MHz, DMSO-*d*_6_) δ 13.00–12.91 (m, 2H, NH–N and NH of indole), 11.81–11.53 (m, 1H, NH–C=O), 8.34 (s, 1H, CH=N), 8.30–8.25 (m, 1H), 7.72–7.66 (m, 4H), 7.54 (d, *J* = 8.3 Hz, 2H), 7.30 (d, *J* = 8.7 Hz, 2H), 7.25–7.16 (m, 3H), 7.01–6.96 (m, 1H), 6.73 (s, 1H), 5.48 (s, 2H, CH_2_–C=O), 5.34 (s, 2H, O-CH_2_); ^13^C NMR (76 MHz, DMSO-*d*_6_) δ 167.70, 167.66, 159.15, 155.62, 146.38, 144.68, 143.12, 139.65, 136.97, 134.92, 132.98, 132.52, 130.52, 129.63, 127.17, 126.62, 126.40, 122.24, 121.64, 121.28, 116.36, 115.17, 113.68, 62.01, 50.93; Anal Calcd for C_27_H_22_ClN_7_O_3_, C, 61.42, H, 4.20, N, 18.57; Found: C, 61.44, H, 4.27, N, 18.51.

### (*E*)-2-(4-((4-((2-(1*H*-indole-2-carbonyl)hydrazono)methyl)phenoxy)methyl)-1*H*-1,2,3-triazol-1-yl)-*N*-(4-chlorophenyl)acetamide (11f)

White solid; isolated yield: 91%, mp: 174–176 °C; IR (KBr, υ): 3281, 3059, 1667 cm^−1^; ^1^H NMR (301 MHz, DMSO-*d*_6_) δ 11.87–11.62 (m, 2H, NH–N and NH of indole), 10.67 (s, 1H, NH–C=O), 8.45 (s, 1H, CH=N), 8.33 (s, 1H), 7.75 (d, *J* = 8.5 Hz, 2H), 7.71–7.59 (m, 3H), 7.50 (d, *J* = 8.2 Hz, 1H), 7.42 (d, *J* = 8.8 Hz, 2H), 7.35 (s, 1H), 7.26 (d, *J* = 7.0 Hz, 1H), 7.19 (d, *J* = 8.3 Hz, 2H), 7.09 (t, *J* = 7.7 Hz, 1H), 5.41 (s, 2H, CH_2_–C=O), 5.29 (s, 2H, O–CH_2_); ^13^C NMR (76 MHz, DMSO-*d*_6_) δ 164.86, 160.07, 158.02, 147.47, 142.75, 137.83, 137.29, 130.69, 129.33, 129.16, 127.88, 127.70, 127.51, 126.90, 125.99, 124.25, 122.21, 120.41, 115.60, 112.86, 103.89, 61.65, 56.53, 21.25; Anal Calcd for C_27_H_22_ClN_7_O_3_, C, 61.42, H, 4.20, N, 18.57; Found: C, 61.44, H, 4.18, N, 18.63.

### (*E*)-2-(4-((4-((2-(1*H*-indole-2-carbonyl)hydrazono)methyl)phenoxy)methyl)-1*H*-1,2,3-triazol-1-yl)-*N*-(4-bromophenyl)acetamide (11g)

White solid; isolated yield: 83%, mp: 239–241 °C; IR (KBr, υ): 3285, 3088, 1645 cm^−1^; ^1^H NMR (301 MHz, DMSO-*d*_6_) δ 11.92–11.61 (m, 2H, NH–N and NH of indole), 10.67 (s, 1H, NH–C=O), 8.50–8.42 (m, 1H, CH=N), 8.33 (s, 1H), 7.78–7.68 (m, 3H), 7.62–7.50 (m, 5H), 7.39–7.33 (m, 1H), 7.26 (d, *J* = 7.0 Hz, 1H), 7.23–7.17 (m, 2H), 7.10 (t, *J* = 7.5 Hz, 1H), 5.42 (s, 2H, CH_2_–C=O), 5.29 (s, 2H, O-CH_2_); ^13^C NMR (76 MHz, DMSO-*d*_6_) δ 164.88, 162.77, 160.08, 158.05, 147.51, 142.76, 138.24, 137.29, 132.23, 130.70, 129.16, 127.71, 127.53, 126.89, 124.27, 122.22, 121.68, 120.42, 115.94, 115.60, 112.87, 61.67, 52.75; Anal Calcd for C_27_H_22_BrN_7_O_3_, C, 56.65, H, 3.87, N, 17.13; Found: C, 56.64, H, 3.80, N, 17.15.

### (*E*)-2-(4-((4-((2-(1*H*-indole-2-carbonyl)hydrazono)methyl)phenoxy)methyl)-1*H*-1,2,3-triazol-1-yl)-*N*-(3-methyl-4-nitrophenyl)acetamide (11h)

White solid; isolated yield: 82%, mp: 200–202 °C; IR (KBr, υ): 3291, 3057, 1668 cm^−1^; ^1^H NMR (301 MHz, DMSO-*d*_6_) δ 11.91–11.63 (m, 2H, NH–N and NH of indole), 10.12 (s, 1H, NH–C=O), 8.48 (s, 1H, CH=N), 8.36 (s, 1H), 8.15 (d, *J* = 2.7 Hz, 1H), 8.08 (dd, *J* = 8.9, 2.7 Hz, 1H), 8.00 (d, *J* = 9.0 Hz, 1H), 7.78–7.72 (m, 2H), 7.62 (d, *J* = 8.1 Hz, 1H), 7.55–7.50 (m, 1H), 7.42–7.35 (m, 1H), 7.29–7.23 (m, 1H), 7.21 (d, *J* = 6.6 Hz, 1H), 7.12 (d, *J* = 4.7 Hz, 1H), 7.10–7.04 (m, 1H), 5.60 (s, 2H, CH_2_–C=O), 5.29 (s, 2H, O–CH_2_), 2.43 (s, 3H, CH_3_); ^13^C NMR (76 MHz, DMSO-*d*_6_) δ 167.66, 160.07, 158.12, 145.48, 143.94, 142.83, 142.58, 138.65, 137.31, 129.17, 128.72, 127.71, 127.54, 126.95, 126.01, 125.98, 124.28, 120.83, 120.43, 115.57, 115.16, 112.88, 104.04, 61.65, 52.72, 21.22; Anal Calcd for C_28_H_24_N_8_O_5_, C, 60.86, H, 4.38, N, 20.28; Found: C, 60.88, H, 4.45, N, 20.25.

### (*E*)-2-(4-((4-((2-(1*H*-indole-2-carbonyl)hydrazono)methyl)-2-methoxyphenoxy)methyl)-1*H*-1,2,3-triazol-1-yl)-*N*-phenylacetamide (11i)

White solid; isolated yield: 91%, mp: 204–206 °C; IR (KBr, υ): 3286, 3069, 1614 cm^−1^; ^1^H NMR (301 MHz, DMSO-*d*_6_) δ 11.91–11.70 (m, 2H, NH–N and NH of indole), 10.53 (s, 1H, NH–C=O), 8.48–8.42 (m, 1H, CH=N), 8.33 (s, 1H), 7.71 (d, *J* = 8.0 Hz, 1H), 7.63 (d, *J* = 7.2 Hz, 2H), 7.51 (d, *J* = 8.3 Hz, 1H), 7.44–7.40 (m, 1H), 7.40–7.33 (m, 3H), 7.33–7.27 (m, 2H), 7.26–7.22 (m, 1H), 7.14–7.07 (m, 2H), 5.41 (s, 2H, CH_2_–C=O), 5.26 (s, 2H, O–CH_2_), 3.85 (s, 3H, O–CH_3_); ^13^C NMR (76 MHz, DMSO-*d*_6_) δ 164.66, 158.03, 149.92, 149.78, 147.91, 142.69, 138.89, 137.30, 130.67, 129.40, 127.96, 127.51, 127.03, 124.27, 122.23, 120.43, 119.72, 113.50, 112.88, 108.93, 103.96, 62.06, 55.90, 52.73; Anal Calcd for C_28_H_25_N_7_O_4_, C, 64.24, H, 4.81, N, 18.73; Found: C, 64.27, H, 4.80, N, 18.78.

### (*E*)-2-(4-((4-((2-(1*H*-indole-2-carbonyl)hydrazono)methyl)-2-methoxyphenoxy)methyl)-1*H*-1,2,3-triazol-1-yl)-*N*-(4-methoxyphenyl)acetamide (11j)

White solid; isolated yield: 92%, mp: 214–216 °C; IR (KBr, υ): 3290, 3054, 1669 cm^−1^; ^1^H NMR (301 MHz, DMSO-*d*_6_) δ 12.02–11.62 (m, 2H, NH–N and NH of indole), 10.70 (s, 1H, NH–C=O), 8.53–8.39 (m, 1H, CH=N), 8.32 (s, 1H), 7.70 (d, *J* = 7.9 Hz, 1H), 7.62–7.50 (m, 6H), 7.43 (d, *J* = 4.5 Hz, 1H), 7.40–7.33 (m, 1H), 7.31–7.28 (m, 1H), 7.24 (d, *J* = 7.8 Hz, 1H), 7.09 (t, *J* = 7.4 Hz, 1H), 5.42 (s, 2H, CH_2_–C=O), 5.26 (s, 2H, O–CH_2_), 3.84 (s, 6H, O–CH_3_); ^13^C NMR (76 MHz, DMSO-*d*_6_) δ 164.10, 156.03, 153.36, 149.91, 149.77, 138.54, 131.97, 130.38, 128.70, 127.98, 127.37, 127.15, 126.32, 126.02, 122.22, 121.28, 120.42, 114.48, 113.65, 112.99, 112.87, 110.15, 108.86, 62.17, 62.08, 55.93, 55.89; Anal Calcd for C_29_H_27_N_7_O_5_, C, 62.92, H, 4.92, N, 17.71; Found: C, 62.95, H, 4.97, N, 17.68.

### (*E*)-2-(4-((4-((2-(1*H*-indole-2-carbonyl)hydrazono)methyl)-2-methoxyphenoxy)methyl)-1*H*-1,2,3-triazol-1-yl)-*N*-(4-chlorophenyl)acetamide (11k)

White solid; isolated yield: 88%, mp: 184–186 °C; IR (KBr, υ): 3293, 3051, 1668 cm^−1^; ^1^H NMR (301 MHz, DMSO-*d*_6_) δ 11.91–11.63 (m, 2H, NH–N and NH of indole), 10.66 (s, 1H, NH–C=O), 8.57–8.37 (m, 1H, CH=N), 8.33 (s, 1H), 7.72–7.63 (m, 3H), 7.52 (d, *J* = 8.2 Hz, 1H), 7.45–7.34 (m, 4H), 7.33–7.21 (m, 3H), 7.09 (t, *J* = 7.5 Hz, 1H), 5.42 (s, 2H, CH_2_–C=O), 5.26 (s, 2H, O–CH_2_), 3.85 (s, 3H, O–CH_3_); ^13^C NMR (76 MHz, DMSO-*d*_6_) δ 164.87, 162.77, 158.05, 149.91, 149.78, 147.92, 140.66, 137.83, 137.30, 130.69, 129.32, 127.98, 127.90, 127.52, 127.07, 124.28, 122.62, 122.23, 120.43, 113.52, 112.88, 108.97, 103.99, 62.07, 52.72, 36.24; Anal Calcd for C_28_H_24_ClN_7_O_4_, C, 60.27, H, 4.34, N, 17.57; Found: C, 60.20, H, 4.37, N, 17.59.

### (*E*)-2-(4-((4-((2-(1*H*-indole-2-carbonyl)hydrazono)methyl)-2-methoxyphenoxy)methyl)-1*H*-1,2,3-triazol-1-yl)-*N*-(2,3-dichlorophenyl)acetamide (11l)

White solid; isolated yield: 91%, mp: 181–183 °C; IR (KBr, υ): 3287, 3051, 1668 cm^−1^; ^1^H NMR (301 MHz, DMSO-*d*_6_) δ 11.87–11.59 (m, 2H, NH–N and NH of indole), 10.29 (s, 1H, NH–C=O), 8.49–8.43 (m, 1H, CH=N), 8.34 (s, 1H), 7.79–7.69 (m, 4H), 7.54–7.48 (m, 2H), 7.37–7.32 (m, 1H), 7.27–7.22 (m, 1H), 7.19 (d, *J* = 8.4 Hz, 2H), 7.13–7.07 (m, 1H), 5.55 (s, 2H, CH_2_–C=O), 5.29 (s, 2H, O–CH_2_), 3.88 (s, 3H, O–CH_3_); ^13^C NMR (76 MHz, DMSO-*d*_6_) δ 165.56, 160.07, 158.06, 147.51, 142.80, 137.29, 136.63, 132.51, 132.26, 130.69, 129.16, 128.62, 128.59, 127.71, 127.55, 126.93, 126.01, 125.49, 124.91, 124.27, 122.21, 120.43, 115.60, 112.87, 103.94, 61.65, 52.48, 21.25; Anal Calcd for C_28_H_23_Cl_2_N_7_O_4_, C, 56.77, H, 3.91, N, 16.55; Found: C, 56.80, H, 3.95, N, 16.56.

### (*E*)-2-(4-((4-((2-(1*H*-indole-2-carbonyl)hydrazono)methyl)-2-methoxyphenoxy)methyl)-1*H*-1,2,3-triazol-1-yl)-*N*-(3,5-dichlorophenyl)acetamide (11m)

White solid; isolated yield: 90%, mp: 247–249 °C; IR (KBr, υ): 3282, 3056, 1668 cm^−1^; ^1^H NMR (301 MHz, DMSO-*d*_6_) δ 11.88–11.69 (m, 2H, NH–N and NH of indole), 10.87 (s, 1H, NH–C=O), 8.48–8.39 (m, 1H, CH=N), 8.33 (s, 1H), 7.75–7.68 (m, 1H), 7.67 (d, *J* = 1.9 Hz, 2H), 7.52 (d, *J* = 8.3 Hz, 1H), 7.42 (s, 1H), 7.39–7.34 (m, 1H), 7.33 (t, *J* = 1.9 Hz, 1H), 7.32–7.26 (m, 2H), 7.24 (d, *J* = 8.5 Hz, 1H), 7.09 (t, *J* = 7.5 Hz, 1H), 5.45 (s, 2H, CH_2_–C=O), 5.27 (s, 2H, O–CH_2_), 3.85 (s, 3H, O–CH_3_); ^13^C NMR (76 MHz, DMSO-*d*_6_) δ 165.56, 158.06, 149.91, 149.78, 147.90, 142.78, 141.14, 137.30, 134.74, 130.67, 128.67, 127.98, 127.52, 127.03, 124.28, 123.56, 122.23, 120.43, 117.93, 113.50, 112.88, 108.95, 103.99, 62.05, 55.90, 52.74; Anal Calcd for C_28_H_23_Cl_2_N_7_O_4_, C, 56.77, H, 3.91, N, 16.55; Found: C, 56.70, H, 3.94, N, 16.59.

### (*E*)-2-(4-((4-((2-(1*H*-indole-2-carbonyl)hydrazono)methyl)-2-methoxyphenoxy)methyl)-1*H*-1,2,3-triazol-1-yl)-*N*-(4-bromophenyl)acetamide (11n)

White solid; isolated yield: 86%, mp: 172–174 °C; IR (KBr, υ): 3289, 3056, 1665 cm^−1^; ^1^H NMR (301 MHz, DMSO-*d*_6_) δ 11.97–11.60 (m, 2H, NH–N and NH of indole), 10.70 (s, 1H, NH–C=O), 8.53–8.38 (m, 1H, CH=N), 8.32 (s, 1H), 7.70 (d, *J* = 7.9 Hz, 1H), 7.64–7.51 (m, 6H), 7.43 (d, *J* = 4.5 Hz, 1H), 7.37 (s, 1H), 7.29–7.27 (m, 1H), 7.26–7.19 (m, 1H), 7.09 (t, *J* = 7.4 Hz, 1H), 5.42 (s, 2H, CH_2_–C=O), 5.26 (s, 2H, O–CH_2_), 3.84 (s, 3H, O–CH_3_); ^13^C NMR (76 MHz, DMSO-*d*_6_) δ 164.90, 158.05, 149.78, 147.92, 138.26, 137.29, 130.66, 130.39, 127.98, 127.51, 127.20, 127.02, 126.32, 124.29, 122.22, 120.45, 120.42, 115.93, 110.17, 108.94, 104.02, 62.06, 55.90, 52.75; Anal Calcd for C_28_H_24_BrN_7_O_4_, C, 55.82, H, 4.02, N, 16.28; found: C, 55.80, H, 4.07, N, 16.23.

### (*E*)-2-(4-((4-((2-(1*H*-indole-2-carbonyl)hydrazono)methyl)-2-methoxyphenoxy)methyl)-1*H*-1,2,3-triazol-1-yl)-*N*-(2-chloro-3-nitrophenyl)acetamide (11o)

White solid; isolated yield: 89%, mp > 250 °C; IR (KBr, υ): 3282, 3056, 1663 cm^−1^; ^1^H NMR (301 MHz, DMSO-*d*_6_) δ 11.88–11.68 (m, 2H, NH–N and NH of indole), 10.28 (s, 1H, NH–C=O), 8.53–8.37 (m, 1H, CH=N), 8.33 (s, 1H), 7.79–7.72 (m, 2H), 7.70 (d, *J* = 8.0 Hz, 1H), 7.53–7.46 (m, 2H), 7.41 (s, 1H), 7.37–7.31 (m, 1H), 7.31–7.26 (m, 2H), 7.26–7.20 (m, 1H), 7.12–7.06 (m, 1H), 5.50 (s, 2H, CH_2_–C=O), 5.26 (s, 2H, O–CH_2_), 3.86 (s, 3H, O–CH_3_); ^13^C NMR (76 MHz, DMSO-*d*_6_) δ 165.51, 157.99, 151.40, 149.90, 149.77, 147.90, 142.81, 137.79, 137.27, 130.70, 130.29, 127.98, 127.50, 127.30, 127.03, 126.97, 125.98, 124.26, 122.20, 121.67, 120.42, 113.54, 112.87, 108.98, 103.94, 62.06, 56.52, 55.92; Anal Calcd for C_28_H_23_ClN_8_O_6_, C, 55.77, H, 3.84, N, 18.58; Found: C, 55.79, H, 3.80, N, 18.57.

### In vitro α‐glucosidase inhibition assay and kinetic study

In vitro α‐glucosidase inhibition evaluations of the indole-carbohydrazide linked phenoxy-1,2,3-triazole-*N*-phenylacetamide derivatives **11a–o** and kinetic study of the most potent compounds **11d**, **11n**, and **11b** were performed on yeast α‐glucosidase according to literature [21].

### Molecular modeling

Molecular modeling of the most potent compounds **11d** and **11n** in the active site of modeled α-glucosidase was carried out according to our previously described method [21]. Yeast form of α-glucosidase, *Saccharomyces cerevisiae,* that was used in in vitro assessments had not any crystallographic structure in the protein data bank, thus, our research team reconstructed a modeled enzyme using SWISS-MODEL Repository [24]. For this purpose, we used of a method that was described by Imran et al. [25, 26]. After searching by using SWISS-MODEL to find an appropriate enzyme with a high sequence similarity with *Saccharomyces cerevisiae* α-glucosidase in protein data bank, we selected *Saccharomyces cerevisiae* isomaltase with code of 3A4A in this bank. *Saccharomyces cerevisiae* isomaltase has 72% identical and 85% similarity with the *Saccharomyces cerevisiae* α-glucosidase. Next, *Saccharomyces cerevisiae isomaltase* was subjected through sequence alignment and homology model was reconstructed using by automated homology modeling pipeline SWISS-MODEL and the quality of the obtained constructed model was verified using PROCHECK [24].

The 3D structures of the most potent compounds **11d** and **11n** were built by MarvineSketch 5.8.3, 2012, ChemAxon (http://www.chemaxon.com) and converted to pdbqt coordinate using Auto dock Tools. The pdbqt coordinate of the target enzyme (modeled α-glucosidase) was created using Auto dock Tools. The obtained pdbqt file of the modeled α-glucosidase was used as an input file for the AUTOGRID program. In this program for each atom type in the title ligands, maps were calculated with 0.375 Å spacing between grid points and the center of the grid box was placed at x = 12.5825, y = − 7.8955, and z = 12.519 Å. The dimensions of the active site box were set at 40 × 40 × 40 Å. Flexible ligand dockings were accomplished for the title compounds. Each docked system for compounds **11n** and **11d** was carried out by 50 runs of the AUTODOCK search by the Lamarckian genetic algorithm. The best poses of the latter compounds were selected for analyzing the interactions between target enzyme and ligands. The results were visualized using BIOVIA Discovery Studio v.3.5 and the obtained data showed in Fig. 6 and Table 2.

### In vitro cytotoxicity assay

Evaluation of cytotoxic effects of the synthesized compounds **11d** and **11n** was performed exactly based on our previous report [22].

### In silico druglikeness/ADME/T studies

In silico druglikeness/ADME/T predictions of the positive control acarbose and the most potent compounds **11d** and **11n** were performed using the preADMET online server [23].

### Statistical analyses

Statistical analysis was carried out using SPSS Software version 16 (IBM Corporation, Armonk, NY, USA).

## Conclusion

In conclusion, we designed indole-carbohydrazide-phenoxy-1,2,3-triazole-*N*-phenylacetamide hybrids **11a–o** as hybrid analogs of the active pharmacophores which previously have been reported as anti-α-glucosidase agents. Compounds **11a–o** were synthesized in good yields. All of the synthesized compounds showed excellent inhibitory activity against α-glucosidase, more than positive control acarbose. Representatively, compound **11d** with IC_50_ value of 6.31 µM was 118.8 times more potent than acarbose. Kinetic studies of the most potent compounds **11d**, **11n**, and **11b** revealed that these compounds are uncompetitive inhibitors against α‐glucosidase. Molecular modeling of the most potent compounds **11d** and **11n** demonstrated that these compounds interact as well with important amino acids at active site. The latter compounds also have good pharmacokinetic properties as oral active compounds.

## Supplementary Information

**Additional file 1.** Contains the IUPAC names, chemical structures, NMR, and IR spectra of the synthesized molecules.

## Data Availability

The datasets used or analyzed during the current study are available from the corresponding author on reasonable request.

## Abbreviations

BBB: Blood brain barrier
DM: Diabetes mellitus
HDF: Human dermal fibroblast
HIA: Human intestinal absorption
T2DM: Type 2 diabetes
