# Supplementary material for: Indole-carbohydrazide linked phenoxy-1,2,3-triazole-N-phenylacetamide derivatives as potent α-glucosidase inhibitors: design, synthesis, in vitro α-glucosidase inhibition, and computational studies
Source: BMC Chem. 2023 Jun 15;17(1):56. doi: 10.1186/s13065-023-00971-w (PMC10268488; doi:10.1186/s13065-023-00971-w)
Supplement: Supplementary file 1 — Additional file 1. Contains the IUPAC names, chemical structures, NMR, and IR spectra of the synthesized molecules. [file 13065_2023_971_MOESM1_ESM.docx]

**Additional information**

**Indole-carbohydrazide linked phenoxy-1,2,3-triazole-*N*-phenylacetamide derivatives as potent α-glucosidase inhibitors: Design, synthesis, *in vitro* α-glucosidase inhibition, and computational studies**

Mehdi Emadi^1^, Fahimeh Mosavizadeh-Marvest^2^, Ali Asadipour^2^, Yaghoub Pourshojaei^2, 3^*, Samanesadat Hosseini^4^, Somayeh Mojtabavi^5^, Mohammad Ali Faramarzi^5^, Bagher Larijani^6^, Maryam Mohammadi-Khanaposhtani^7^, Mohammad Mahdavi^6^*

^1^Electrical and Computer Engineering Department, Babol Noshirvani University of Technology, Babol, Iran. ^2^Department of Medicinal Chemistry, Faculty of Pharmacy, Kerman University of Medical Sciences, Kerman, Iran. ^3^Extremophile and Productive Microorganisms Research Center, Kerman University of Medical Sciences, Kerman, Iran.^4^Shahid Beheshti University of Medical Sciences, Tehran, Iran. ^5^Department of Pharmaceutical Biotechnology, Faculty of Pharmacy, Tehran University of Medical Sciences, Tehran, Iran. ^6^Endocrinology and Metabolism Research Center, Endocrinology and Metabolism Clinical Sciences Institute, Tehran University of Medical Sciences, Tehran, Iran. ^7^Cellular and Molecular Biology Research Center, Health Research Institute, Babol University of Medical Sciences, Babol, Iran.

*Correspondences:

[pourshojaei@yahoo.com](mailto:pourshojaei@yahoo.com)

Department of Medicinal Chemistry, Faculty of Pharmacy, Kerman University of Medical Sciences, Kerman, Iran, and Extremophile and Productive Microorganisms Research Center, Kerman University of Medical Sciences, Kerman, Iran.

[momahdavi@tums.ac.ir](mailto:momahdavi@tums.ac.ir)

Endocrinology and Metabolism Research Center, Endocrinology and Metabolism Clinical Sciences Institute, Tehran University of Medical Sciences, Tehran, Iran.

***(E)-2-(4-((4-((2-(1H-indole-2-carbonyl)hydrazono)methyl)phenoxy)methyl)-1H-1,2,3-triazol-1-yl)-N-phenylacetamide (11a)***

*
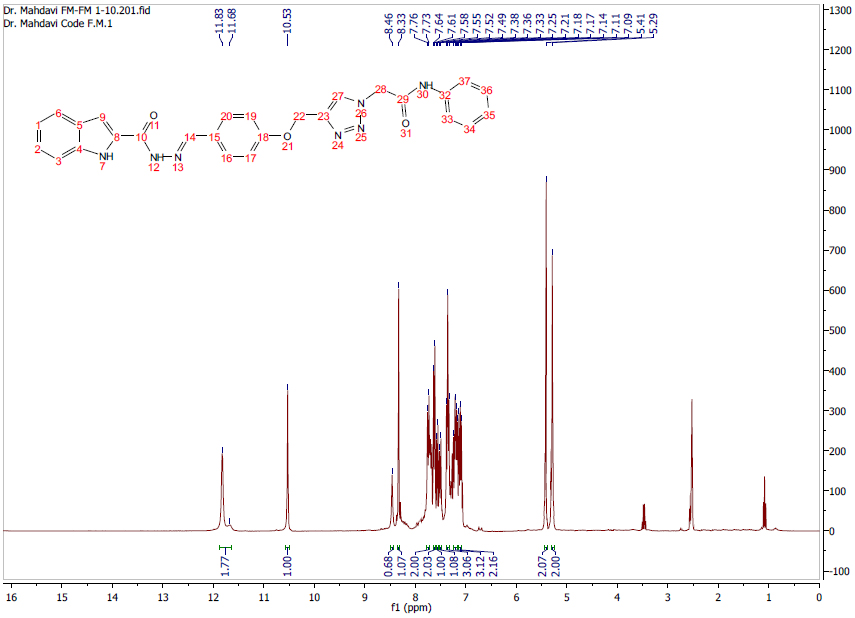
*

**Fig. S1.** ^1^H NMR spectrum of product **11a**

*
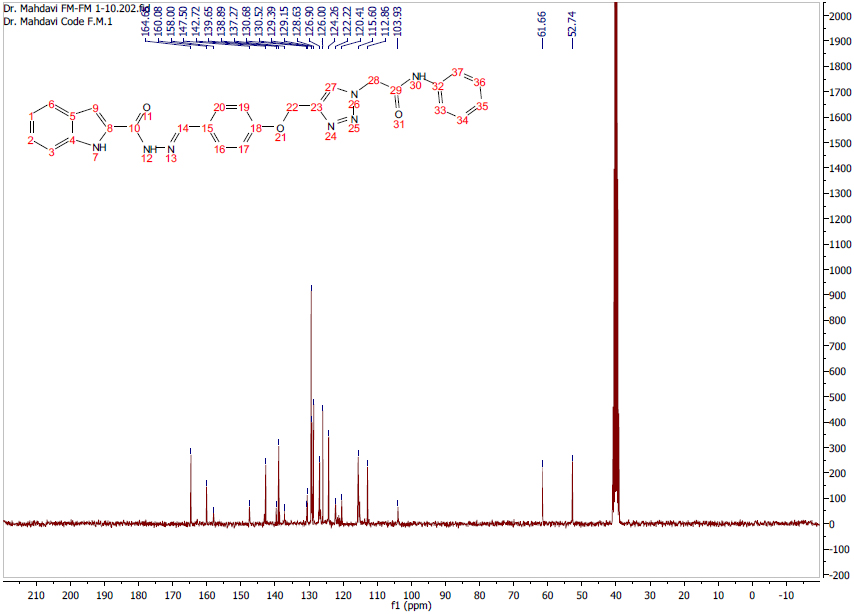
*

**Fig. S2.** ^13^CNMR spectrum of product **11a**

*
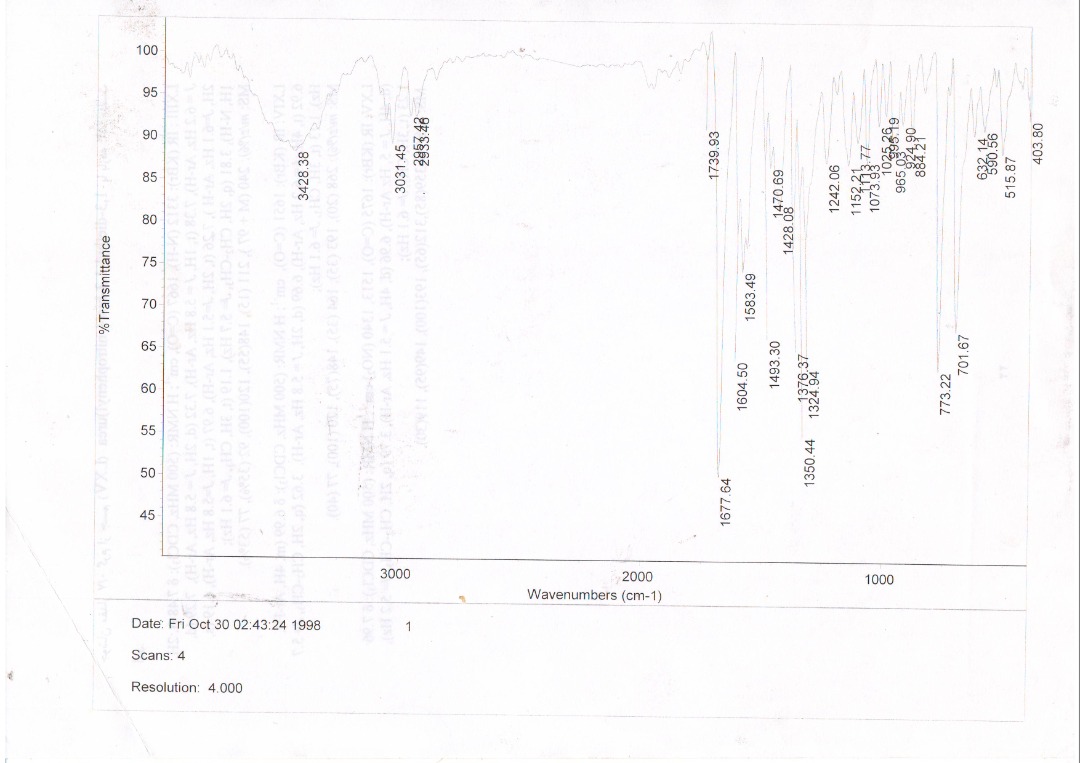
*

**Fig. S3.** IR spectrum of product **11a**

***(E)-2-(4-((4-((2-(1H-indole-2-carbonyl)hydrazono)methyl)phenoxy)methyl)-1H-1,2,3-triazol-1-yl)-N-(o-tolyl)acetamide (11b)***

*
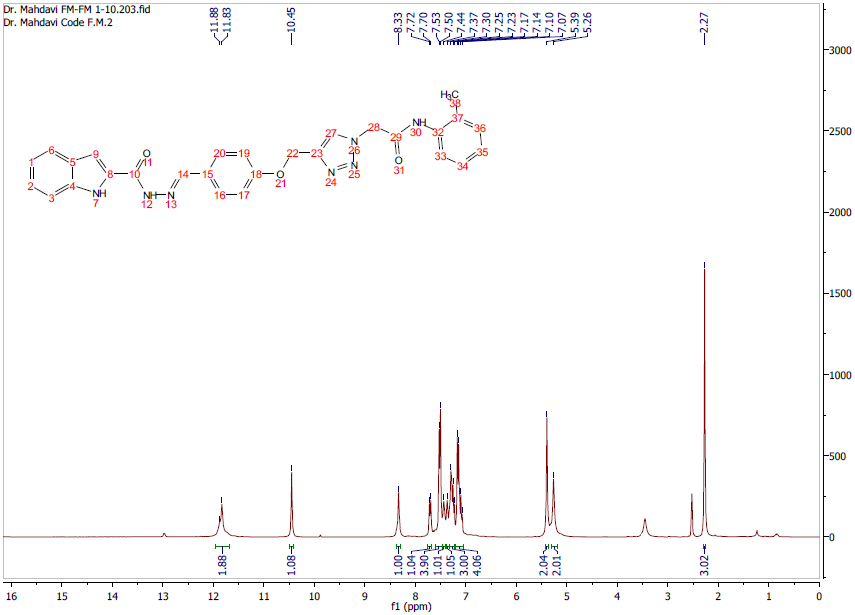
*

**Fig. S4.** ^1^H NMR spectrum of product **11b**

*
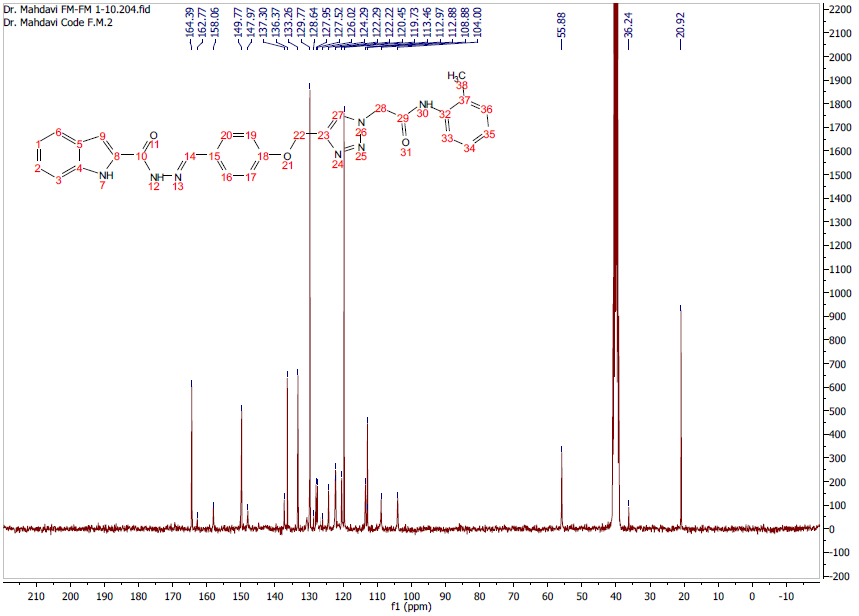
*

**Fig. S5.** ^13^CNMR spectrum of product **11b**

***(E)-2-(4-((4-((2-(1H-indole-2-carbonyl)hydrazono)methyl)phenoxy)methyl)-1H-1,2,3-triazol-1-yl)-N-(p-tolyl)acetamide (11c)***

*
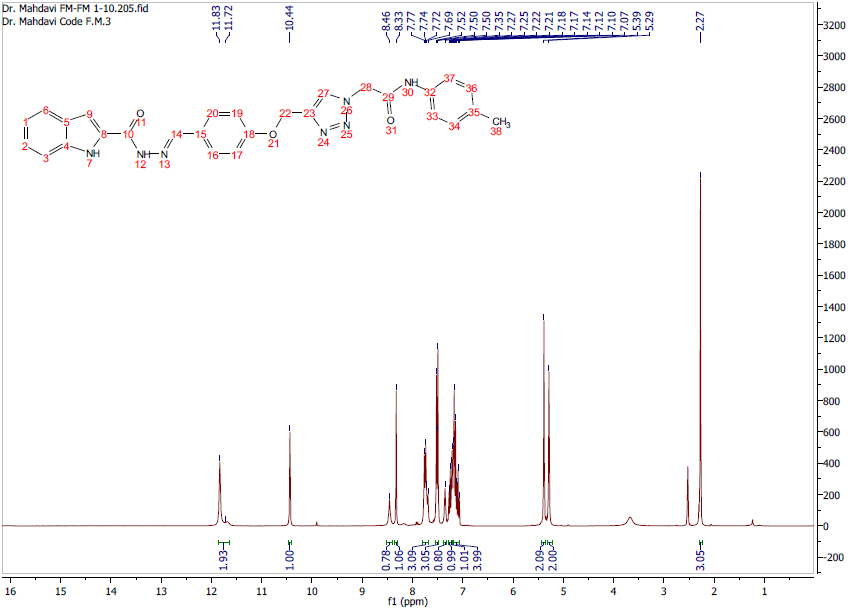
*

**Fig. S6.** ^1^H NMR spectrum of product **11c**

*
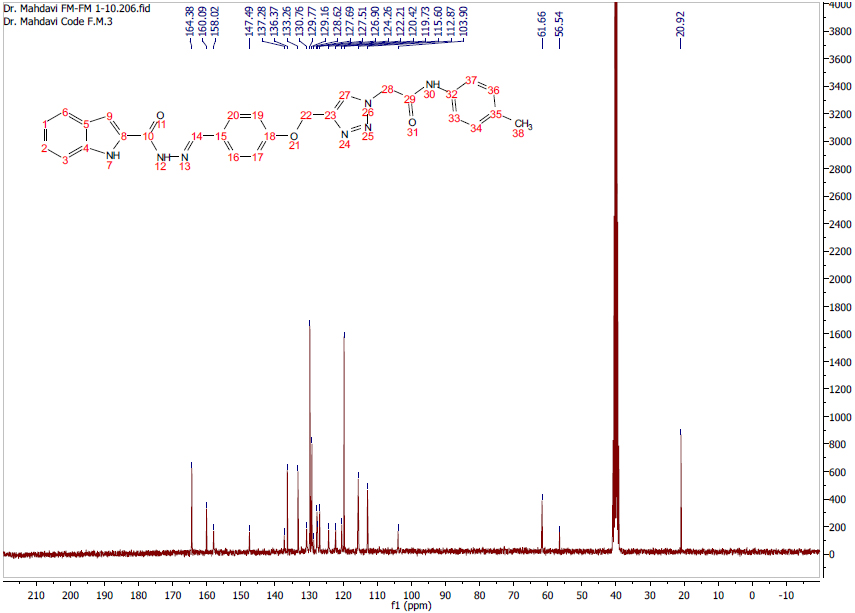
*

**Fig. S7.** ^13^CNMR spectrum of product **11c**

***
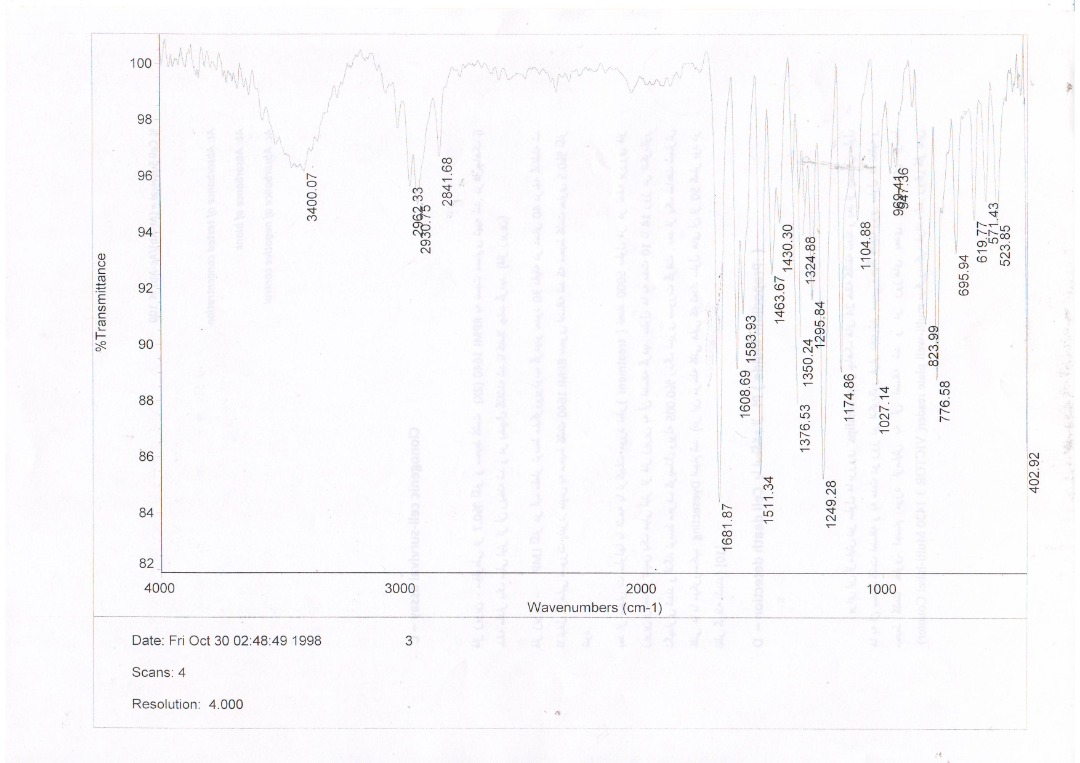
***

**Fig. S8.** IR spectrum of product **11c**

***(E)-2-(4-((4-((2-(1H-indole-2-carbonyl)hydrazono)methyl)phenoxy)methyl)-1H-1,2,3-triazol-1-yl)-N-(4-methoxyphenyl)acetamide (11d)***

*
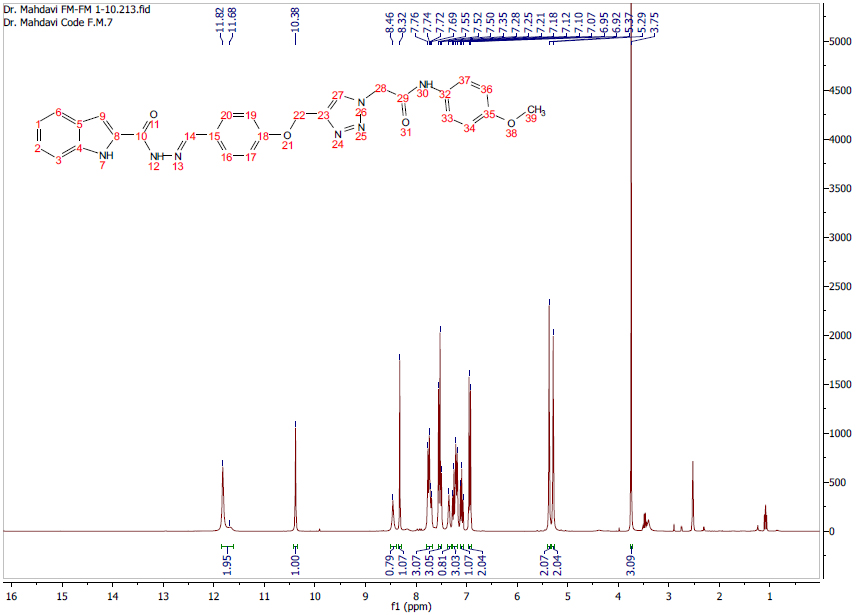
*

**Fig. S9.** ^1^H NMR spectrum of product **11d**

*
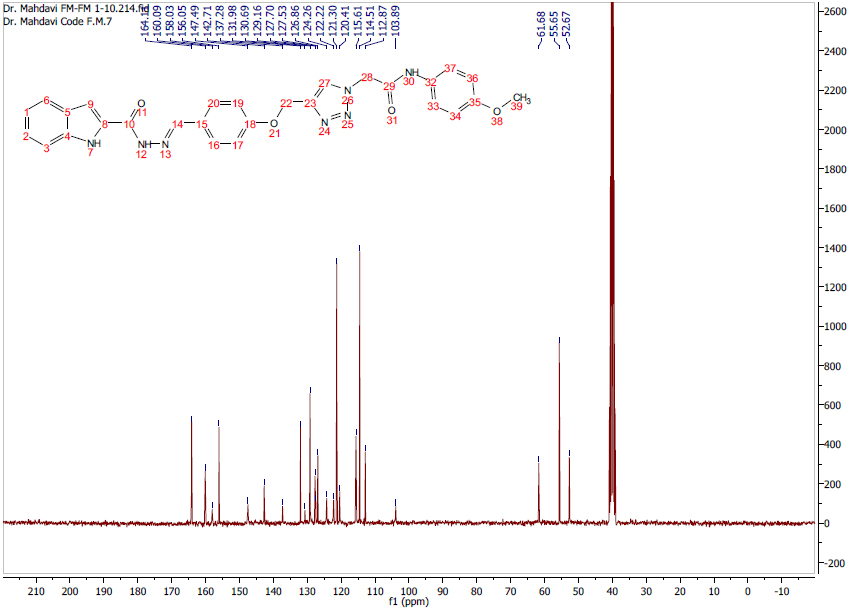
*

**Fig. S10.** ^13^CNMR spectrum of product **11d**

***(E)-2-(4-((4-((2-(1H-indole-2-carbonyl)hydrazono)methyl)phenoxy)methyl)-1H-1,2,3-triazol-1-yl)-N-(2-chlorophenyl)acetamide (11e)***

*
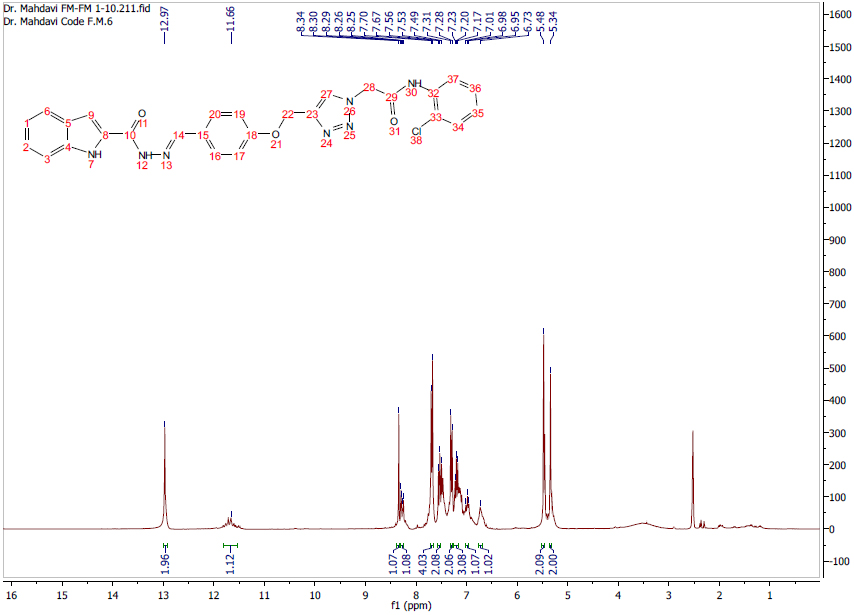
*

**Fig. S11.** ^1^H NMR spectrum of product **11e**

*
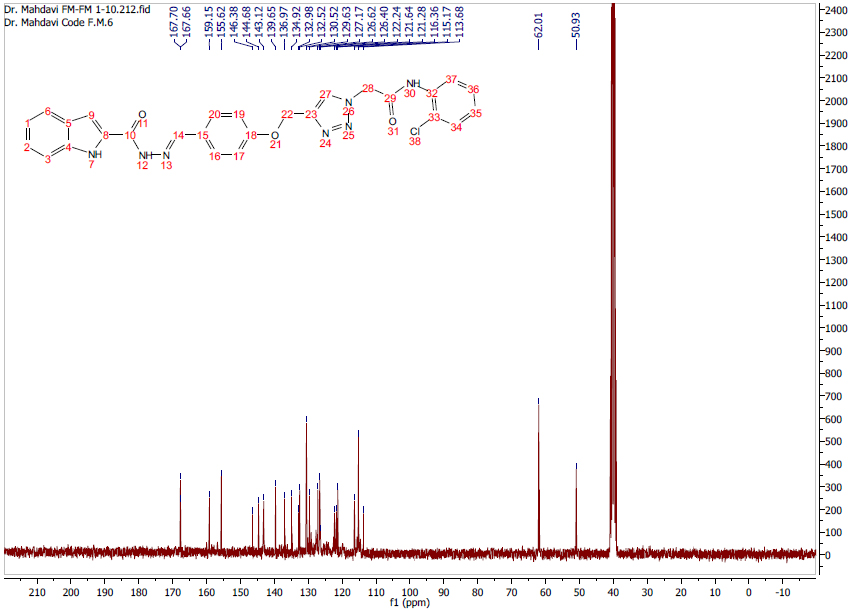
*

**Fig. S12.** ^13^CNMR spectrum of product **11e**

***(E)-2-(4-((4-((2-(1H-indole-2-carbonyl)hydrazono)methyl)phenoxy)methyl)-1H-1,2,3-triazol-1-yl)-N-(4-chlorophenyl)acetamide (11f)***

*
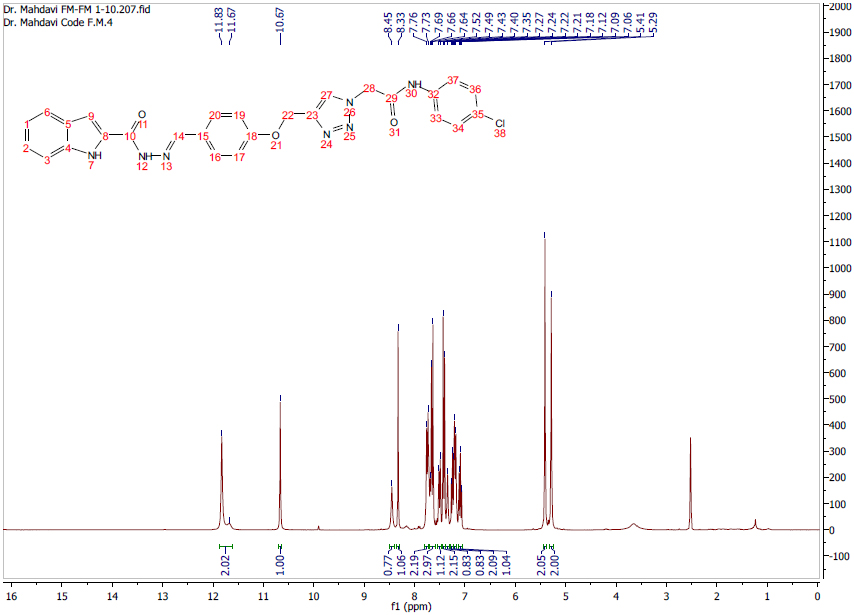
*

**Fig. S13.** ^1^H NMR spectrum of product **11f**

*
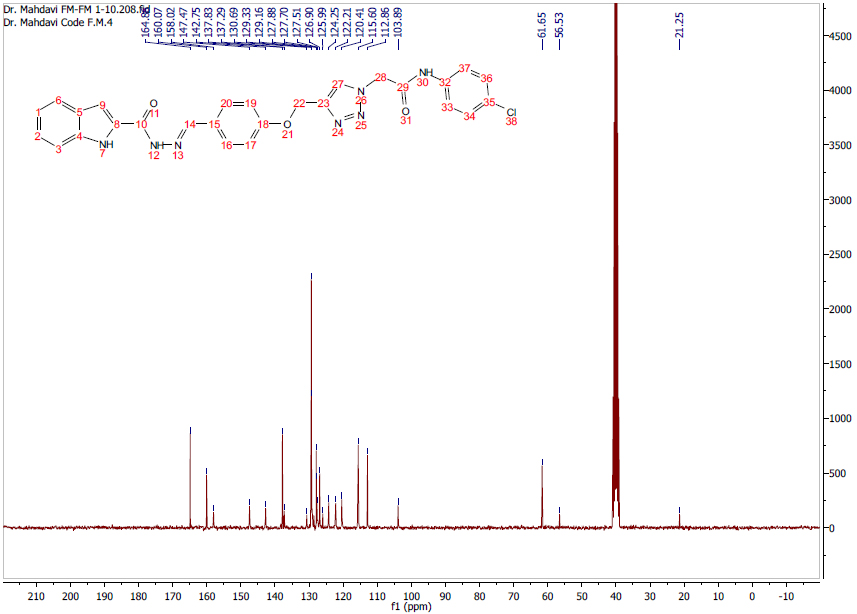
*

**Fig. S14.** ^13^CNMR spectrum of product **11f**

***
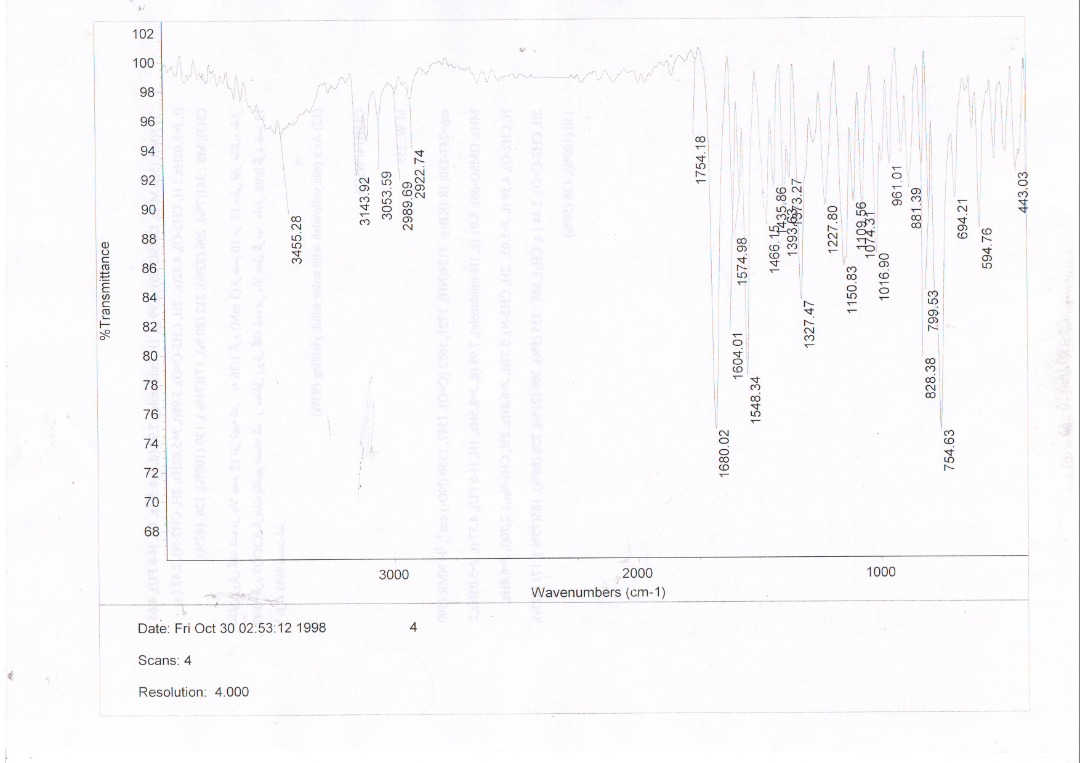
***

**Fig. S15.** IR spectrum of product **11f**

***(E)-2-(4-((4-((2-(1H-indole-2-carbonyl)hydrazono)methyl)phenoxy)methyl)-1H-1,2,3-triazol-1-yl)-N-(4-bromophenyl)acetamide (11g)***

*
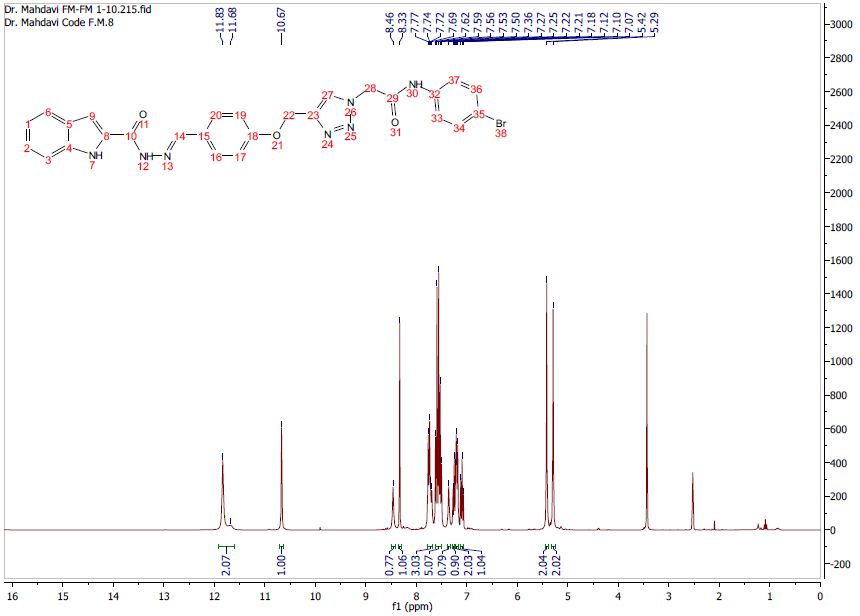
*

**Fig. S16.** ^1^H NMR spectrum of product **11g**

*
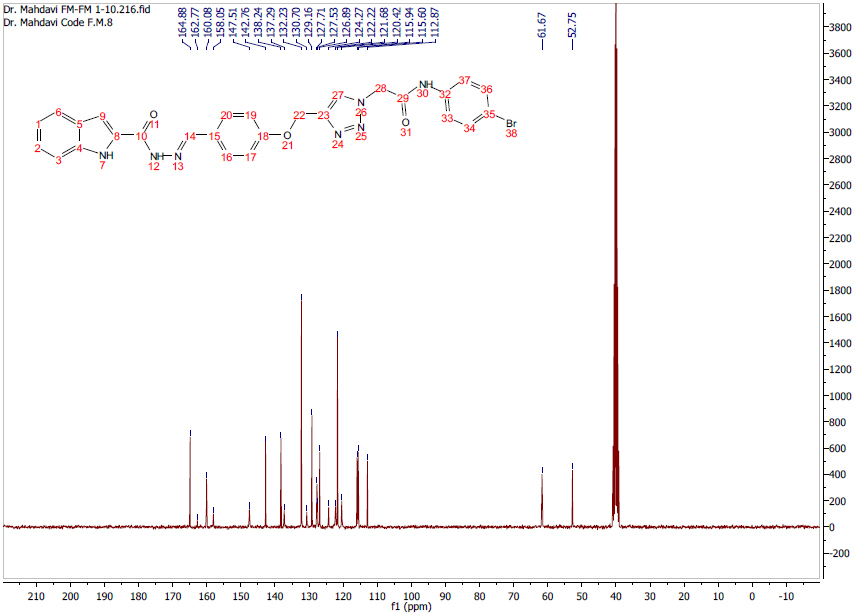
*

**Fig. S17.** ^13^CNMR spectrum of product **11g**

***
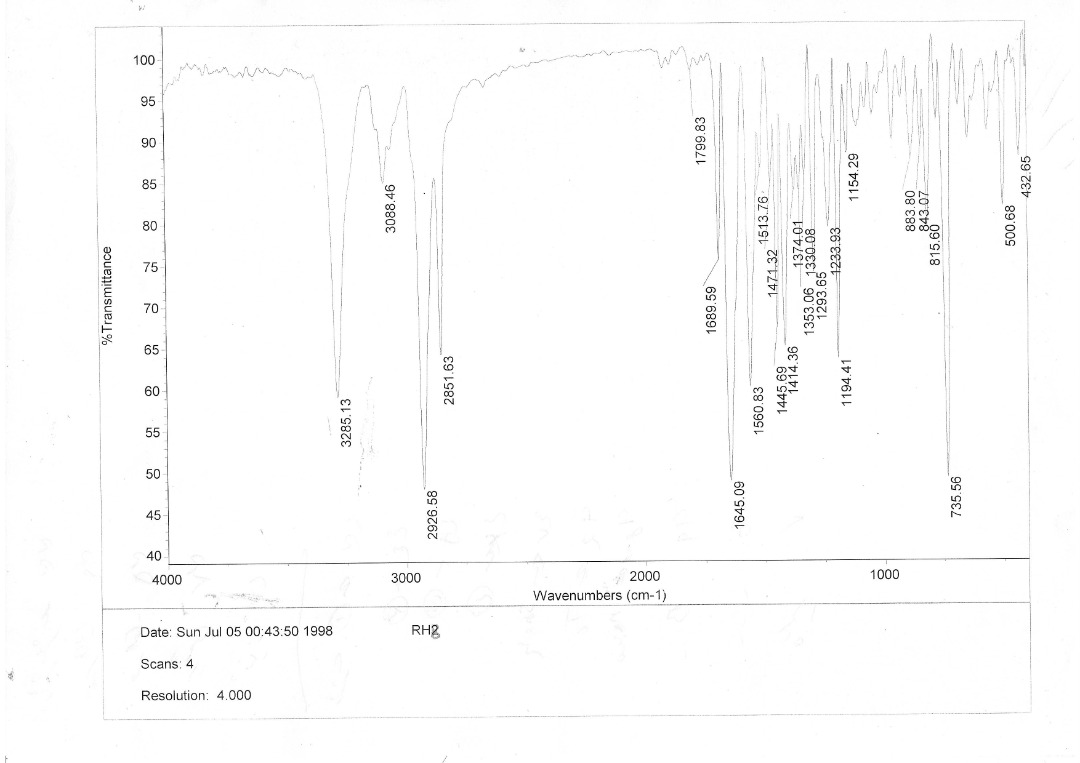
***

**Fig. S18.** IR spectrum of product **11g**

***(E)-2-(4-((4-((2-(1H-indole-2-carbonyl)hydrazono)methyl)phenoxy)methyl)-1H-1,2,3-triazol-1-yl)-N-(3-methyl-4-nitrophenyl)acetamide (11h)***

*
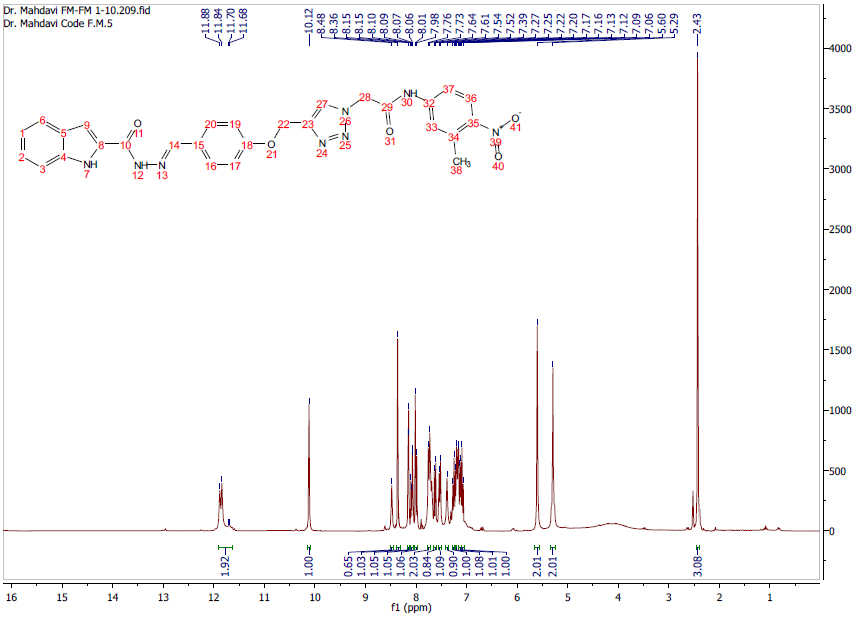
*

**Fig. S19.** ^1^H NMR spectrum of product **11h**

*
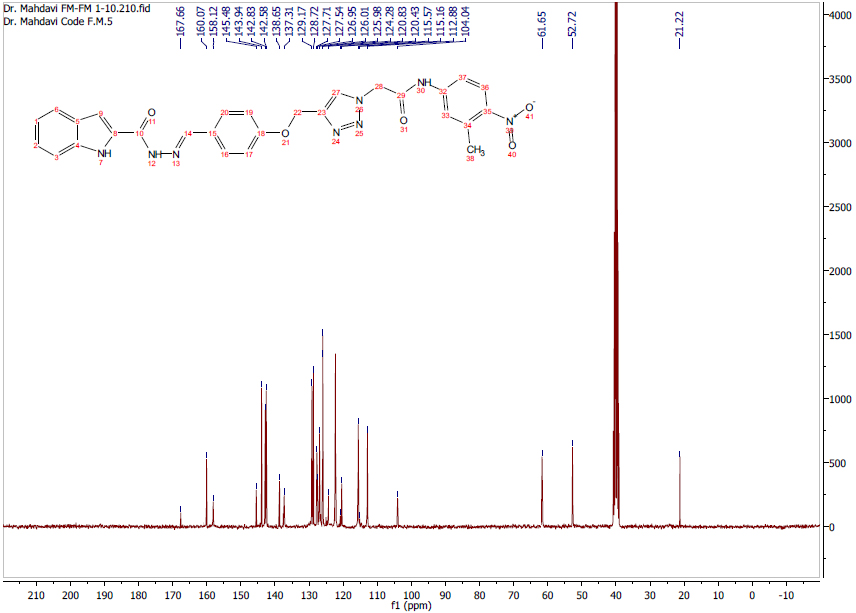
*

**Fig. S20.** ^13^CNMR spectrum of product **11h**

***(E)-2-(4-((4-((2-(1H-indole-2-carbonyl)hydrazono)methyl)-2-methoxyphenoxy)methyl)-1H-1,2,3-triazol-1-yl)-N-phenylacetamide (11i)***

*
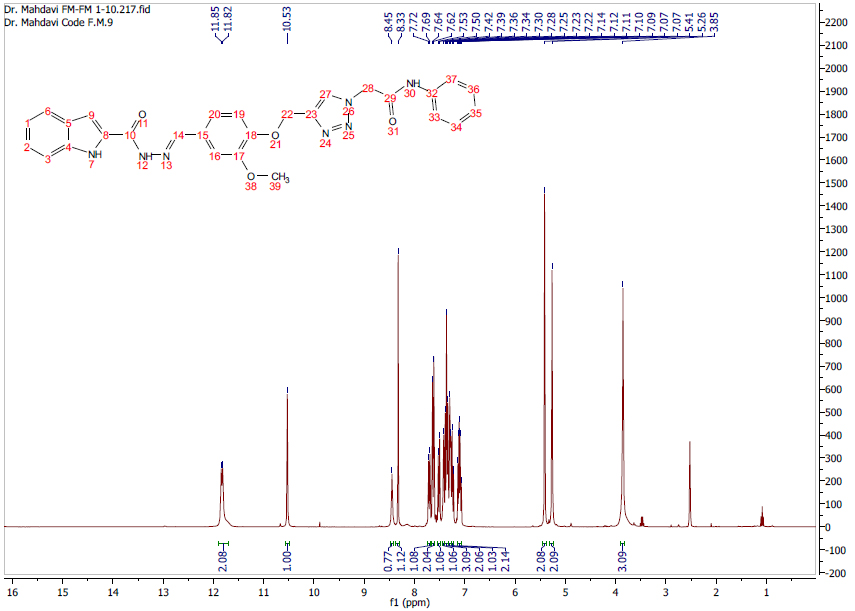
*

**Fig. S21.** ^1^H NMR spectrum of product **11i**

*
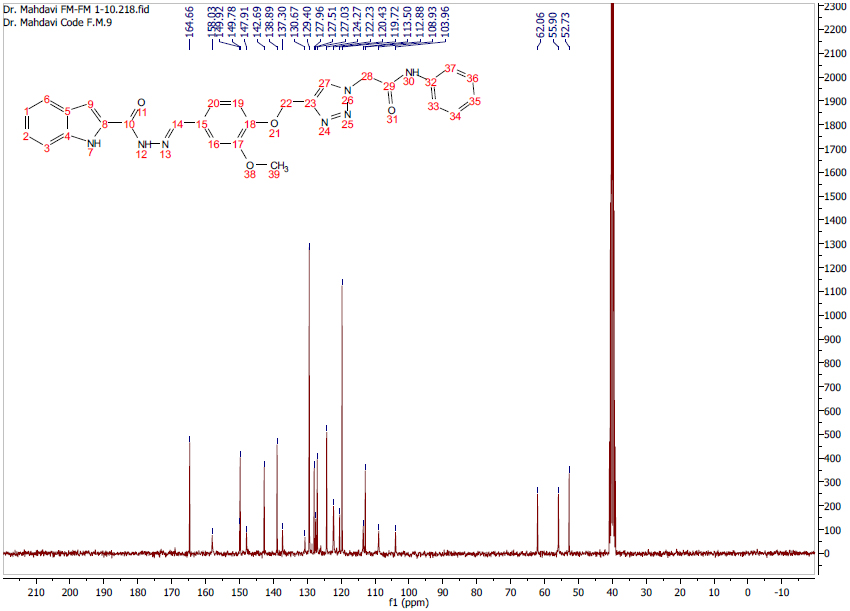
*

**Fig. S22.** ^13^CNMR spectrum of product **11i**

***
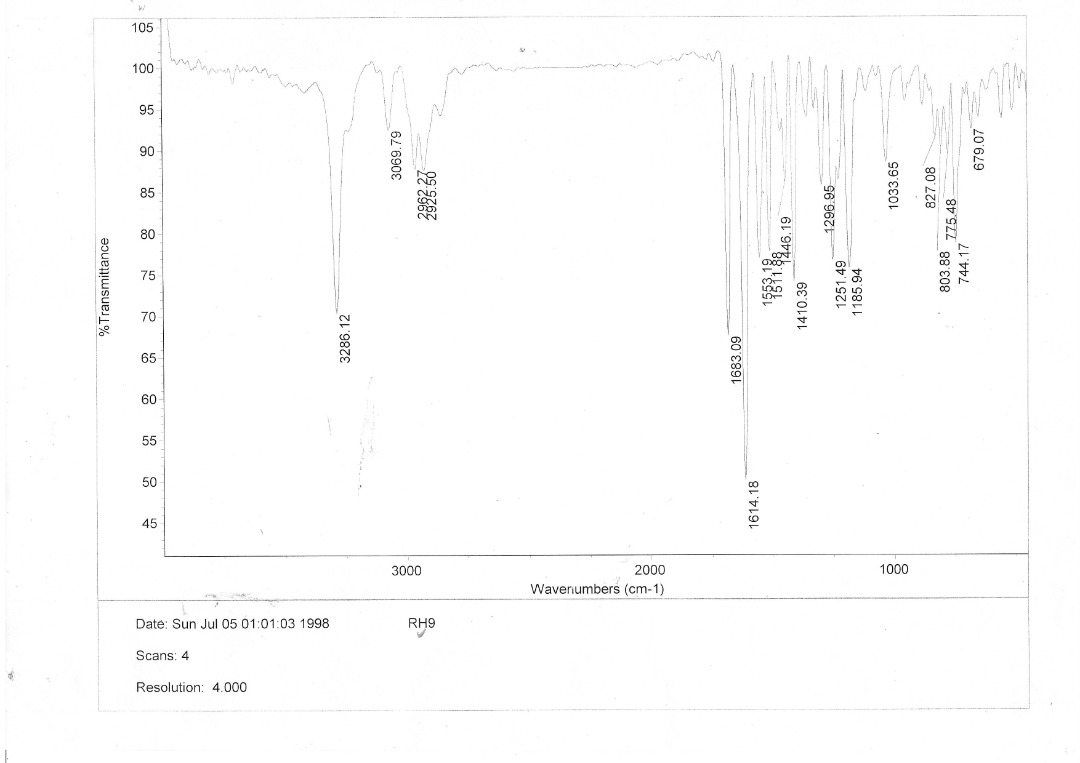
***

**Fig. S23.** IR spectrum of product **11i**

***(E)-2-(4-((4-((2-(1H-indole-2-carbonyl)hydrazono)methyl)-2-methoxyphenoxy)methyl)-1H-1,2,3-triazol-1-yl)-N-(4-methoxyphenyl)acetamide (11j)***

*
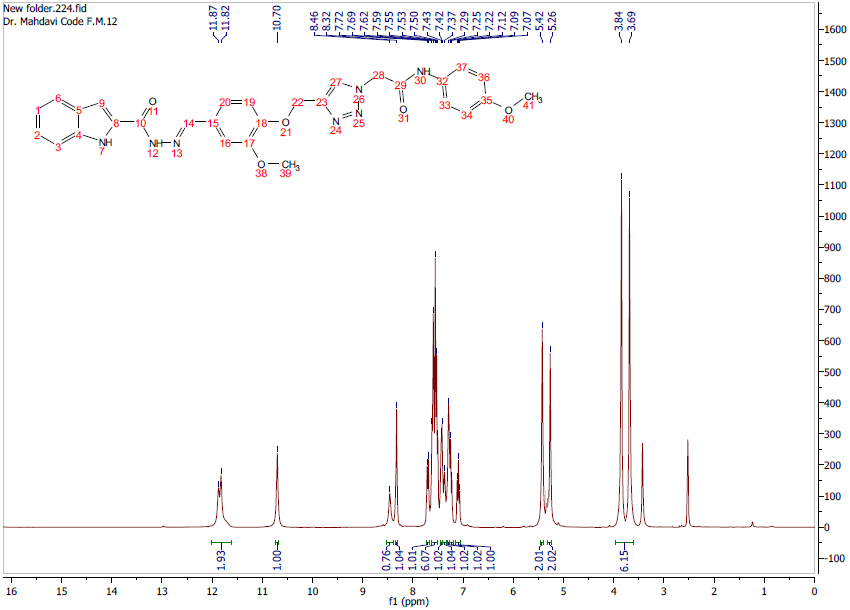
*

**Fig. S24.** ^1^H NMR spectrum of product **11j**

*
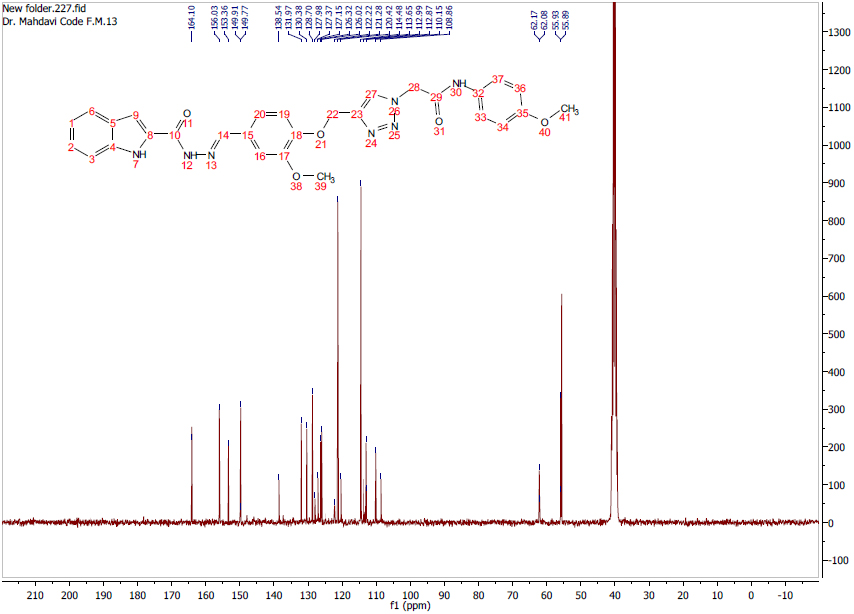
*

**Fig. S25.** ^13^CNMR spectrum of product **11j**

***(E)-2-(4-((4-((2-(1H-indole-2-carbonyl)hydrazono)methyl)-2-methoxyphenoxy)methyl)-1H-1,2,3-triazol-1-yl)-N-(4-chlorophenyl)acetamide (11k)***

*
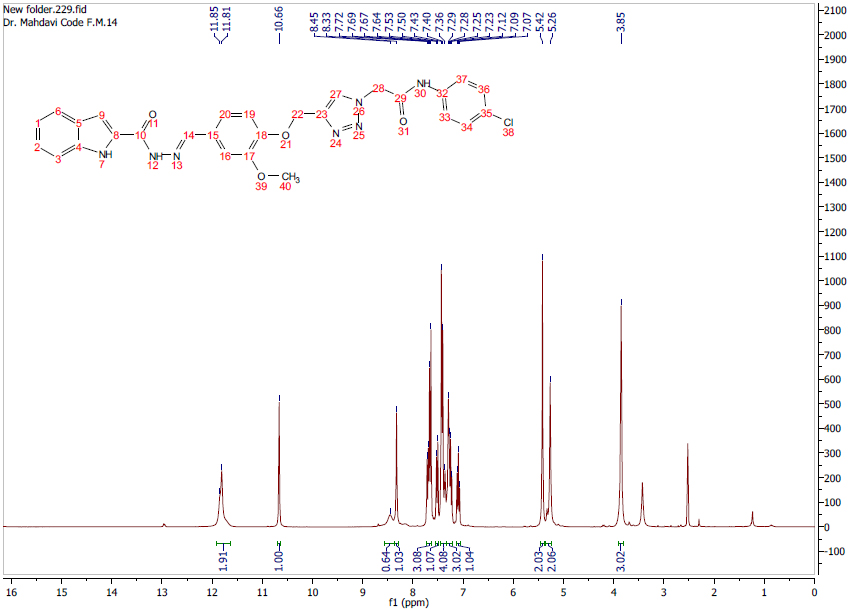
*

**Fig. S26.** ^1^H NMR spectrum of product **11k**

*
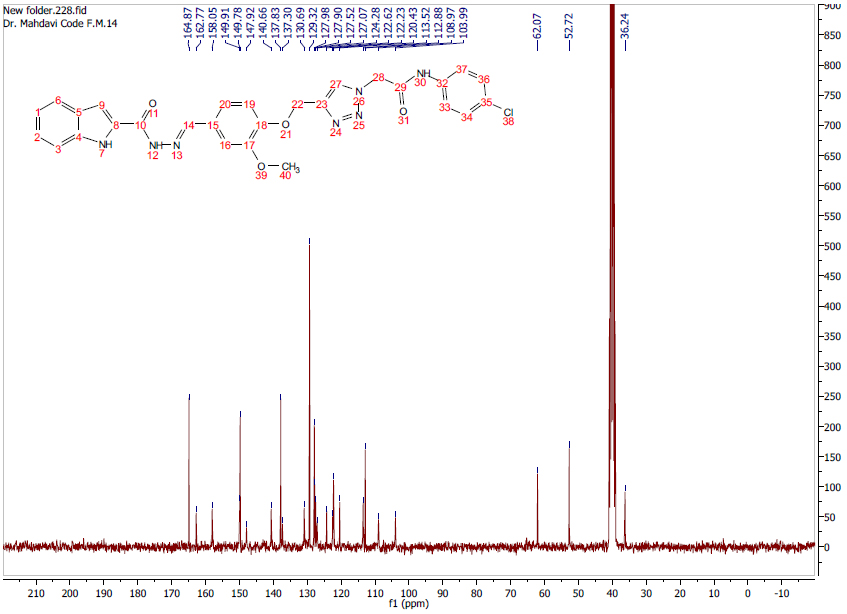
*

**Fig. S27.** ^13^CNMR spectrum of product **11k**

***(E)-2-(4-((4-((2-(1H-indole-2-carbonyl)hydrazono)methyl)-2-methoxyphenoxy)methyl)-1H-1,2,3-triazol-1-yl)-N-(2,3-dichlorophenyl)acetamide (11l)***

*
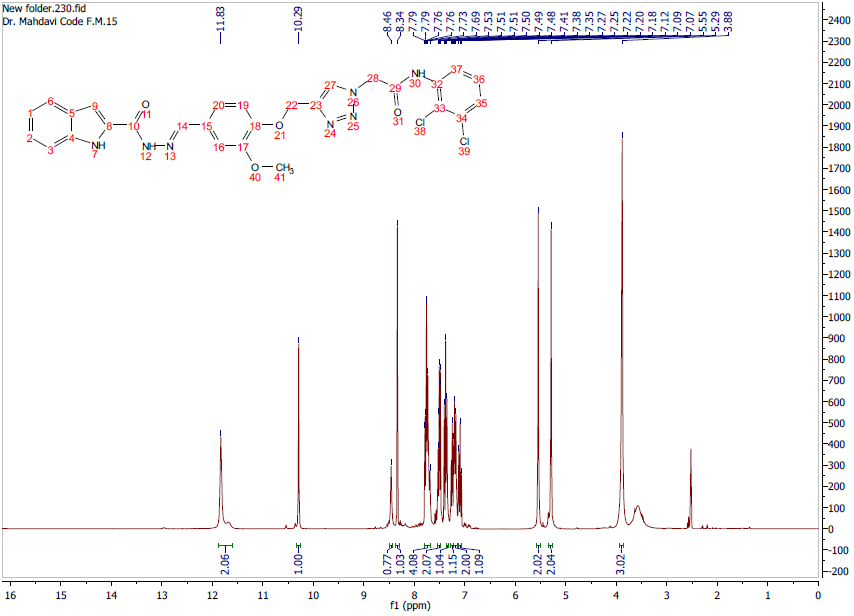
*

**Fig. S28.** ^1^H NMR spectrum of product **11l**

*
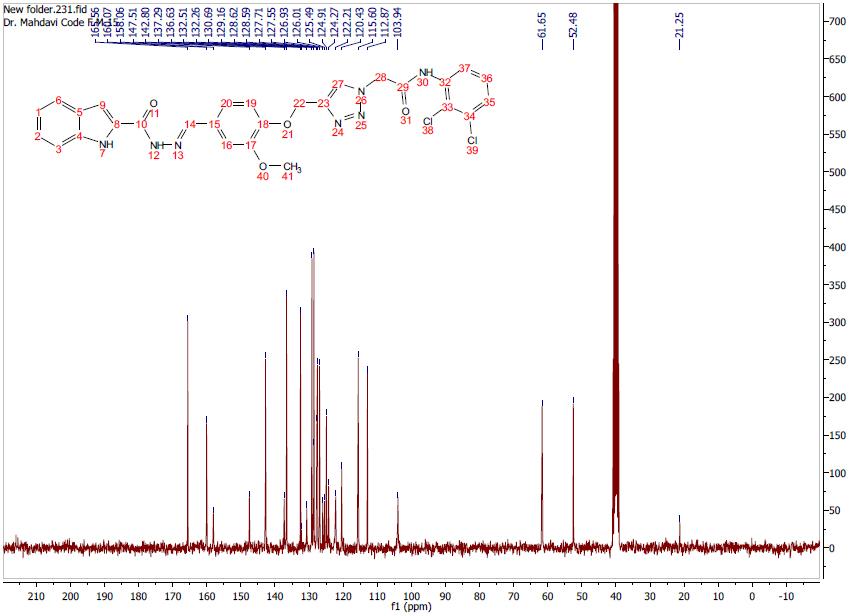
*

**Fig. S29.** ^13^CNMR spectrum of product **11l**

***(E)-2-(4-((4-((2-(1H-indole-2-carbonyl)hydrazono)methyl)-2-methoxyphenoxy)methyl)-1H-1,2,3-triazol-1-yl)-N-(3,5-dichlorophenyl)acetamide (11m)***

*
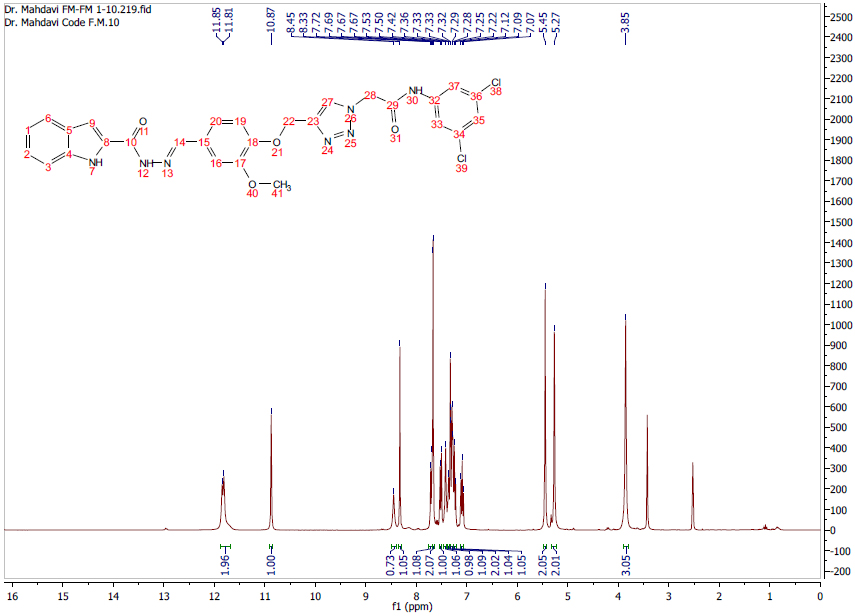
*

**Fig. S30.** ^1^H NMR spectrum of product **11m**

*
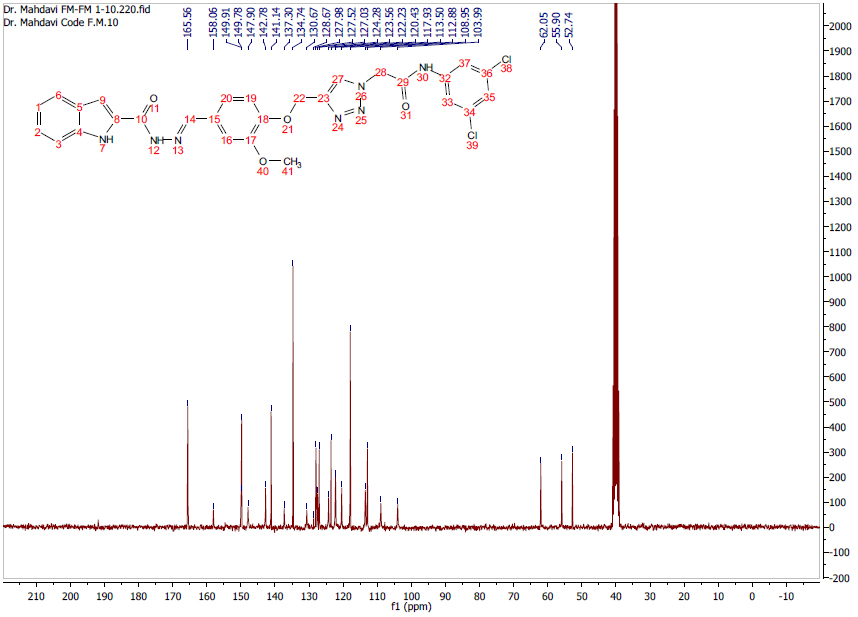
*

**Fig. S31.** ^13^CNMR spectrum of product **11m**

***(E)-2-(4-((4-((2-(1H-indole-2-carbonyl)hydrazono)methyl)-2-methoxyphenoxy)methyl)-1H-1,2,3-triazol-1-yl)-N-(4-bromophenyl)acetamide (11n)***

*
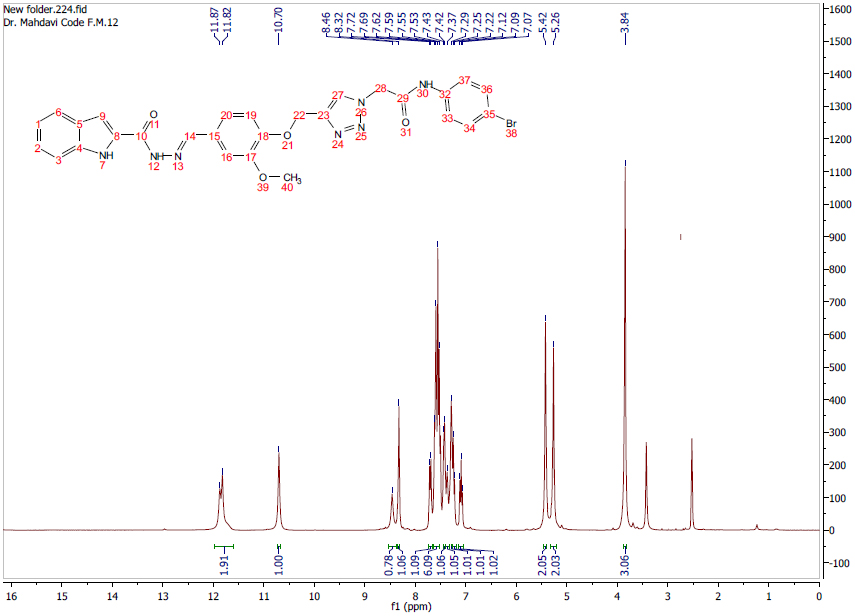
*

**Fig. S32.** ^1^H NMR spectrum of product **11n**

*
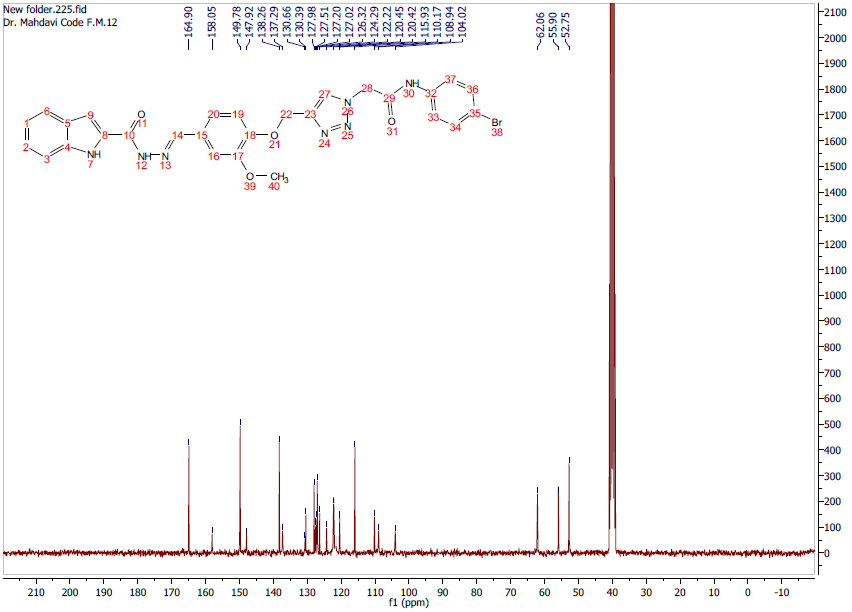
*

**Fig. S33.** ^13^CNMR spectrum of product **11n**

***(E)-2-(4-((4-((2-(1H-indole-2-carbonyl)hydrazono)methyl)-2-methoxyphenoxy)methyl)-1H-1,2,3-triazol-1-yl)-N-(2-chloro-3-nitrophenyl)acetamide (11o)***

*
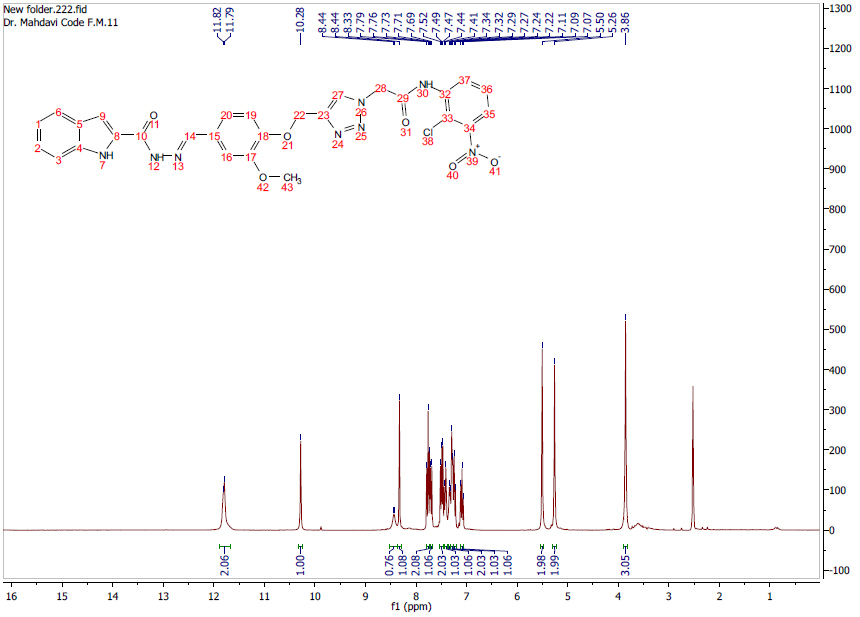
*

**Fig. S34.** ^1^H NMR spectrum of product **11o**

*
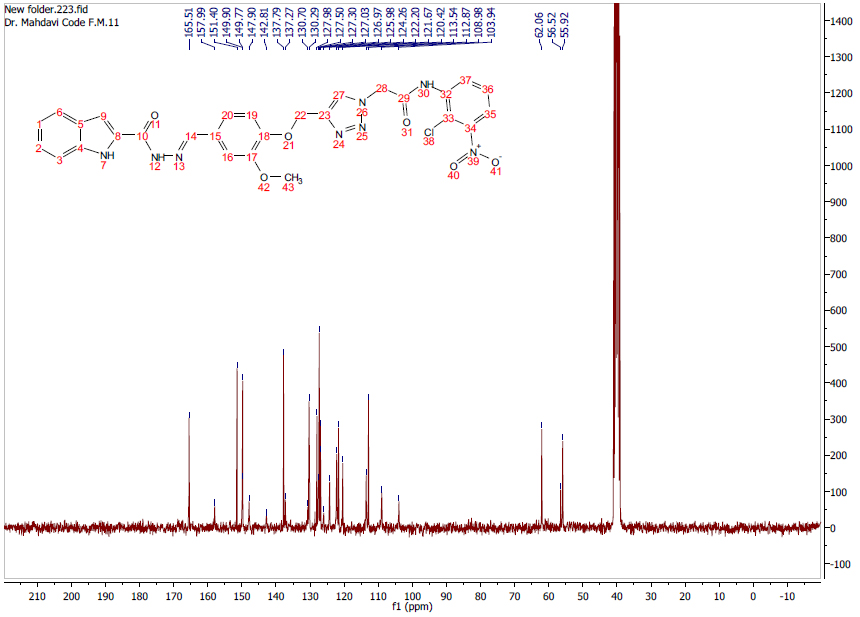
*

**Fig. S35.** ^13^CNMR spectrum of product **11o**
